# Comparative profiling of serum, urine, and feces bile acids in humans, rats, and mice

**DOI:** 10.1038/s42003-024-06321-3

**Published:** 2024-05-27

**Authors:** Dan Zheng, Kun Ge, Chun Qu, Tao Sun, Jieyi Wang, Wei Jia, Aihua Zhao

**Affiliations:** 1 Center for Translational Medicine, Shanghai Key Laboratory of Diabetes Mellitus and Shanghai Key Laboratory of Sleep Disordered Breathing, Shanghai Sixth People’s Hospital Affiliated to Shanghai Jiao Tong University School of Medicine, Shanghai, 200233 China; 2 Department of Pharmacology and Pharmacy, University of Hong Kong, Hong Kong, China

**Keywords:** Differentiation, Metabolomics, Metabolomics, Small molecules

## Abstract

Bile acids (BAs) play important pathophysiological roles in both humans and mammalian animals. Laboratory rats and mice are widely used animal models for assessing pharmacological effects and their underlying molecular mechanisms. However, substantial physiological differences exist in BA composition between humans and murine rodents. Here, we comprehensively compare BA profiles, including primary and secondary BAs, along with their amino acid conjugates, and sulfated metabolites in serum, urine, and feces between humans and two murine rodents. We further analyze the capabilities in gut microbial transform BAs among three species and compare sex-dependent variations within each species. As a result, BAs undergo amidation predominately with glycine in humans and taurine in mice but are primarily unamidated in rats. BA sulfation is a unique characteristic in humans, whereas rats and mice primarily perform multiple hydroxylations during BA synthesis and metabolism. For gut microbial transformed BA capabilities, humans are comparable to those of rats, stronger than those of mice in deconjugation and 7α-dehydroxylation, while humans are weak than those of rats or mice in oxidation and epimerization. Such differences enhance our understanding of the divergent experimental outcomes observed in humans and murine rodents, necessitating caution when translating findings from these rodent species to humans.

## Abstract (web summary)

The analysis of bile acid profiles in biological samples from humans, rats, and mice reveals species-dependent differences as well as sex-dependent differences within each species.

## Introduction

Bile acids (BAs) are a class of steroidal compounds synthesized from cholesterol in the liver. BAs not only play important roles in the digestion and absorption of dietary lipids and hydrophobic vitamins^1^ but also act as signaling molecules that regulate various metabolic pathways by modulating BA nuclear receptors^2,3^. Over the past two decades, research has revealed the involvement of BAs in several pathophysiological pathways, including liver diseases^4,5^, diabetes^6,7^, tumors^8,9^, and Alzheimer’s disease^10,11^. Understanding BA metabolism and function in physiological conditions can help identify therapeutic targets and elucidate the molecular mechanisms underlying these pathological conditions.

Animal experiments are indispensable for bridging the gap between preclinical and clinical studies, and mice and rats are the conventionally used murine rodents in such experiments^12,13^. There have been some reports comparing BA profiles across different animal species^14–16^ or different biological samples within the same species^17–19^. However, these studies lack consideration of biological sex. Results from several studies indicate that sex of both humans and murine rodents impacts the BA synthesis and BA pool composition^20–22^, but their quantification methods are only targeted a few of BAs in plasma. Thus, further investigation is needed to understand the sex differences in BA profiles.

**Fig. 1:**
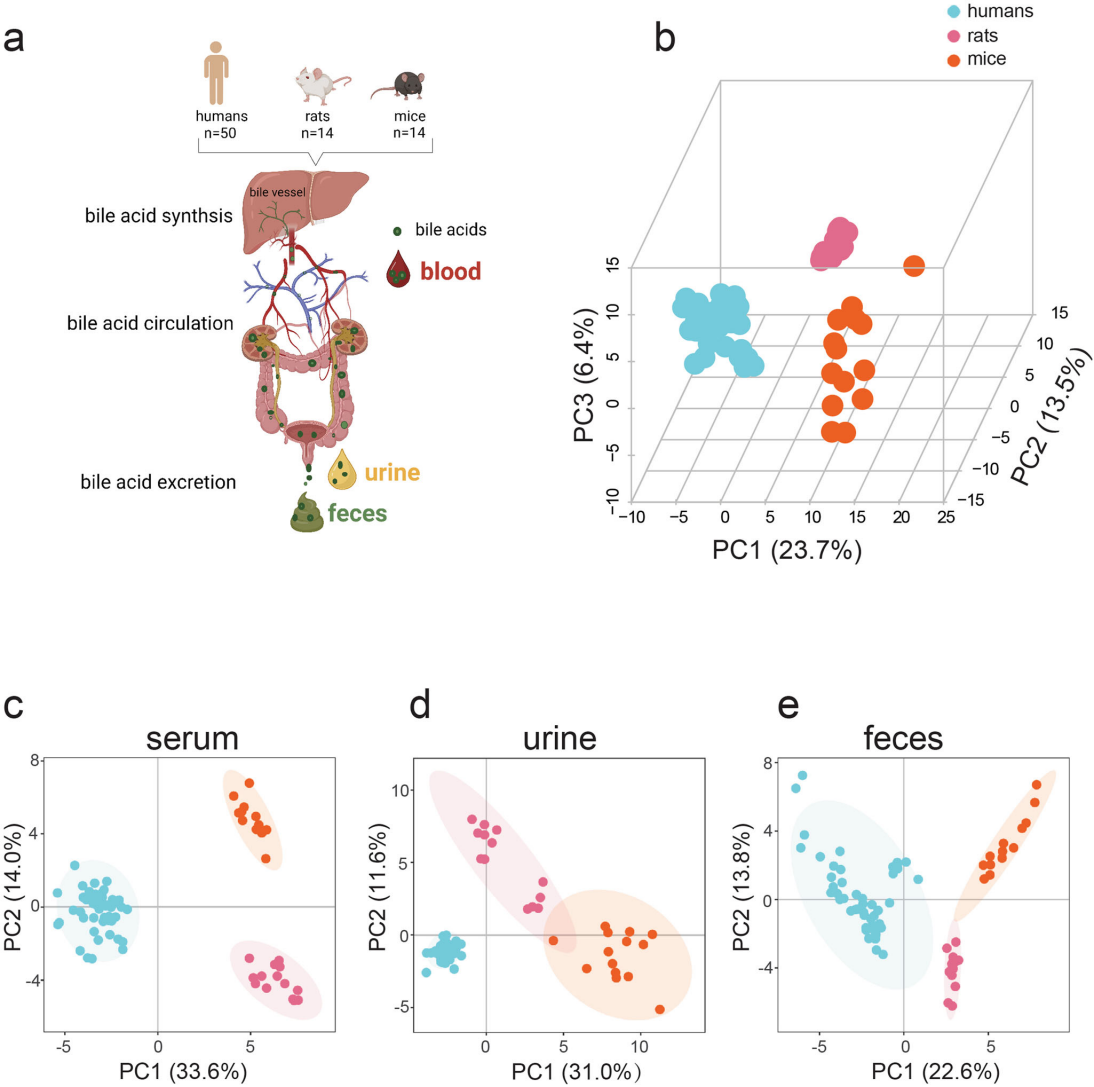
BA workflow and BA differences in humans, rats, and mice. **a** BA synthesis workflow, circulation, and excretion (Created with BioRender.com, with permission). **b** PCA of total BAs in humans, rats, and mice serum, urine, and feces. **c** PCA of serum BAs in humans, rats, and mice. **d** PCA of urine BAs in humans, rats, and mice. **e** PCA of feces BAs in humans, rats, and mice. Human (*n* = 50), rat (*n* = 14), and mice (*n* = 14).

BAs are synthesized in the liver and subsequently undergo a series of metabolic processes. These mainly include phase I metabolism and phase II metabolism, with phash II metabolism typically catalyzed by sulfotransferase, glucuronidase, and glutathione transferase in the liver or intestines. The BA pool in different body fluids or tissues comprises BAs and all their derivatives from phase I and II metabolic processes. BAs and BA derivatives are excreted in feces and urine (Fig. 1a). According to reports, BA glucuronidation in mammals is <0.5%^14^, and sulfation is the prominent metabolism that leads to BA detoxification for excretion in the urine. Before excretion in the feces, BAs undergo a series of microbial transformations catalyzed by the gut microbiota, mainly including deconjugation, 7α-dehydroxylation, oxidation, and epimerization. BA microbial transformation in the gut modify primary BAs (PBAs) into secondary BAs (SBAs) that greatly increase BAs diversity. Despite these known transformations, there have been no reports systematically comparing the differences in BA transformation by gut microbes among humans and murine rodents.

In this study, we have developed an effective method for the quantitative analysis of 65 BAs (Supplementary Table 1), including PBAs and SBAs, along with their amino acid conjugates and sulfated metabolites, in serum, urine, and feces. Subsequently, we systematically present BA profiles in three biological samples from humans, rats, and mice to identify their unique and shared characteristics. Particularly, we comprehensively compare the differences in the capabilities of gut microbe transformation of BAs across species, emphasizing the importance of considering these variations when interpreting and applying the study outcomes across different species.

## Results

### Bile acids differed distinctly among humans, rats, and mice

We collected age-, sex-, and BMI-matched human serum, urine, and fecal samples (*n* = 50) along with age- and sex-matched rat and mouse serum, urine, and feces (*n* = 14 in each animal) to determine the BA and sulfated BA profiles. Not surprisingly, there was a marked difference in the principal component analysis (PCA) among human, rat, and mouse BA profiles (Fig. 1b) when serum, urine, and feces samples were analyzed such that human, rat, and mouse profiles clustered independently. There was also a similarly significant separation when BA variables were analyzed from serum, urine, or feces individually (Fig. 1c–e). These results implied that BA levels and compositions in the serum, urine, and feces samples of humans, rats, and mice differed significantly.

### Serum bile acid profiles of humans, rats, and mice

**Fig. 2:**
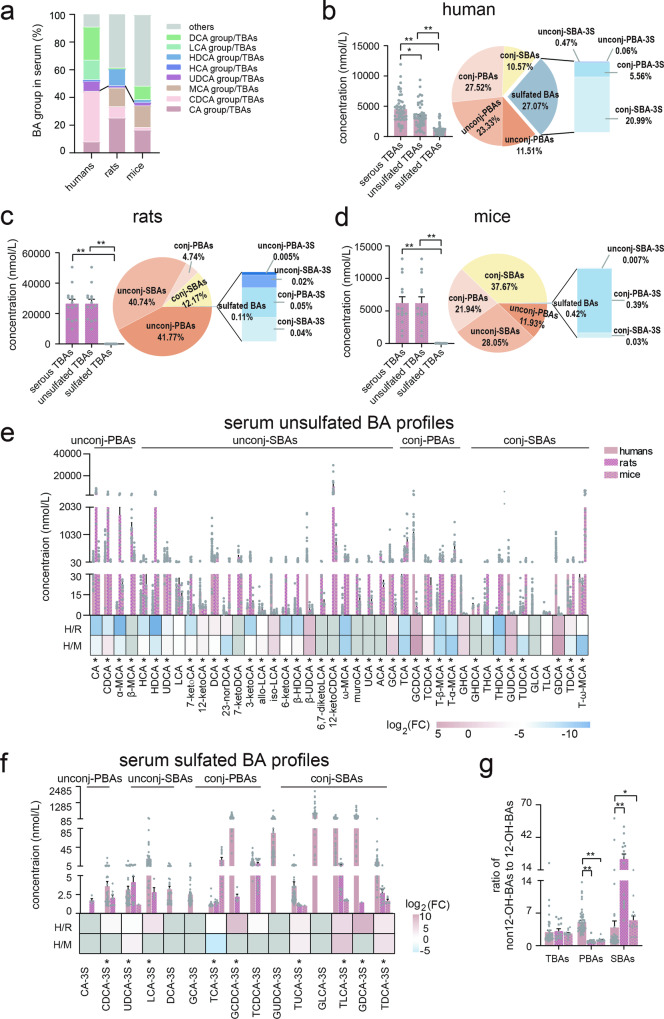
Serum BAs in humans, rats, and mice. **a** The percentage of serum BA groups among humans, rats, and mice. **b** Total BAs and unsulfated/sulfated BA compositions in human serum. **c** Total BA levels and unsulfated/sulfated BA compositions in rat serum. **d** Total BA levels and unsulfated/sulfated BA compositions in mouse serum. **e** Serum unsulfated BA profiles and comparison among humans, rats, and mice. **f** Serum sulfated BA profiles and comparison between humans, rats, and mice. **g** The ratio of serum non-12-OH-BAs to 12-OH-BAs among humans, rats and mice. The colors blue, pink, and gray in heat maps indicate decreased, increased, and below detection limits in at least one group, respectively. Human (*n* = 50), rat (*n* = 14), and mice (*n* = 14). CA group: sum of CA, TCA, GCA, and their sulfated metabolites, CDCA group: sum of CDCA, TCDCA, GCDCA, and their sulfated metabolites, MCA group: sum of α-/β-MCA, and T-α-/β-MCA, UDCA group: sum of UDCA, TUDCA, GUDCA, and their sulfated metabolites, HCA group: sum of HCA, THCA, and GHCA, HDCA group: sum of HDCA, THDCA, and GHDCA, LCA group: sum of LCA, TLCA, GLCA, and their sulfated metabolites, DCA group: sum of DCA, TDCA, GDCA, and their sulfated metabolites, others: excluding above mentioned BA groups. Unconj-PBAs unconjugated primary BAs, unconj-SBAs unconjugated secondary BAs, conj-PBAs conjugated primary BAs, conj-SBAs conjugated secondary BAs, unconj-PBA-3S sulfated unconj-PBA at C-3, unconj-SBA-3S sulfated unconj-SBA at C-3, conj-PBA-3S sulfated PBA at C-3, conj-SBA-3S sulfated conj-SBA at C-3, 12-OH-BAs 12-hydroxylated-BAs. TBAs total BAs, PBAs primary BAs, SBAs secondary BAs. FC fold change of humans to rats or mice, H humans, R rats, M mice, * indicates *p* < 0.05, ** indicates *p* < 0.01. Error bars depict the standard error of the mean (SEM).

Cholic acid (CA), chenodeoxycholic acid (CDCA), along their glycine and taurine conjugates (GCA, TCA, GCDCA, and TCDCA) are classified as PBAs in humans. In rats and mice, CA, CDCA, ursodeoxycholic acid (UDCA), along with their glycine and taurine conjugates, muricholic acid (α-/β-MCA) and MCA taurine conjugates are PBAs. We first represented serum BA general differences among the three species (Fig. 2a). For humans, CDCA group (sum of CDCA, TCDCA, GCDCA, and their sulfated metabolites) was the most predominant portion in PBAs, and deoxycholic acid (DCA) group (sum of DCA, TDCA, GDCA, and their sulfated metabolites) was predominant in SBAs. For rats, CA group was predominant in PBAs, and other BAs (excluding CA group, CDCA group, UDCA group, MCA group, DCA group, LCA group, HCA group, and HDCA group) were predominant in SBAs. For mice, CA group and MCA group (sum of α-/β-MCA, T-α-/β-MCA), were almost comparable in PBAs, others BAs was also predominant in SBAs. Notably, the percentages of PBAs were comparable among the three species. The percentages of DCA group and LCA group were highest in human serum SBAs, while the percentage of HDCA group (sum of HDCA, THDCA, and GHDCA) was highest in rat serum SBAs. When considering total serum BA levels (including unsulfated BAs and sulfated BAs), the level in rats was the highest (26.57 µmol/L), followed by mice (6.22 µmol/L), and then humans (4.51 µmol/L). However, humans had the highest sulfated BA levels of 1.15 µmol/L, accounting for 25.50% of total serum BAs (Fig. 2b). In humans, unconjugated PBAs (unconj-PBAs) and unconjugated SBAs (unconj-SBAs) comprised 11.51% and 23.56% of the total, respectively, whereas conjugated PBAs (conj-PBAs, primary BAs conjugated with glycine or taurine) and conjugated SBAs (conj-SBAs, secondary BAs conjugated with glycine or taurine) comprised 27.52% and 11.05% of the total, respectively. Of the sulfated BAs, conj-PBAs and conj-SBAs accounted for 5.56% and 20.99% respectively, whereas only 0.06% were unconj-PBAs and 0.47% were unconj-SBAs. Despite having the highest serum levels and in stark contrast with humans, sulfated BAs only accounted for 0.11% of total BAs in rats, which contained 0.005% conj-PBAs, 0.02% conj-SBAs, 0.05% unconj-PBAs, and 0.04% unconj-SBAs. Unlike human BA compositions, unconj-PBAs and unconj-SBAs were dominant in rat serum BAs, accounting for 42.24% and 51.65%, respectively, and contained 4.74% conj-PBAs and 12.17% conj-SBAs (Fig. 2c). In mice, 0.39% conj-PBAs, 0.03% conj-SBAs, and 0.001% unconj-PBAs, comprised 0.42% of sulfated BAs (Fig. 2d). Similarly, unsulfated BA profiles in mice serum differed significantly from human serum: conj-PBAs (21.94%) and conj-SBAs (37.67%) were the most abundant types, whereas unconj-PBAs (11.93%) and unconj-SBAs (28.91%) were less prevalent.

In comparison, 36 of the 42 detected unsulfated BAs differed significantly (*p* < 0.05) among human, rat, and mouse serum (Fig. 2e and Supplementary Table 2). 28 BAs differed significantly (*p* < 0.05) between humans and rats, and 24 BAs differed significantly (*p* < 0.05) between humans and mice (Supplementary Table 2). Most humans to rats (H/R) or mice (H/M) BA ratios were decreased in the individual BA heatmap excepting 12-ketoLCA, DCA, iso-LCA, β-UDCA, GCA, GCDCA, TCDCA, GUDCA, and GDCA. Additionally, 9 of the 15 detected sulfated BAs differed significantly (*p* < 0.05) among the three groups (Fig. 2f and Supplementary Table 2), and sulfated BA levels in human serum were higher than those in rats and mice. Notably, a sulfated GLCA (GLCA-3S) was the most abundant sulfated BA in human serum. Overall, the five most abundant BAs in serum by percentage were GCDCA, GLCA-3S, DCA, CDCA, and β-UDCA in humans; 12-ketoCDCA, CA, HDCA, CDCA, and α-MCA in rat; and T-ω-MCA, 12-ketoCDCA, TCA, DCA, and T-β-MCA in mice. 12-Hydroxylated-BAs (12-OH-BAs) with a hydroxy group at C-12 include CA, DCA, and their conjugated, sulfated metabolites. The ratio of non12-OH-BAs (sum of CDCA group, LCA group, UDCA group, MCA group, HCA group, HDCA group, and others) to 12-OH-BAs (sum of CA group and DCA group) was equivalent among the three species (Fig. 2g). However, the ratio of non12-OH-PBAs to 12-OH-PBAs was the highest while the ratio of non12-OH-SBAs to 12-OH-SBAs was the lowest in humans (Fig. 2g).

### Urine bile acid profiles of humans, rats, and mice

**Fig. 3:**
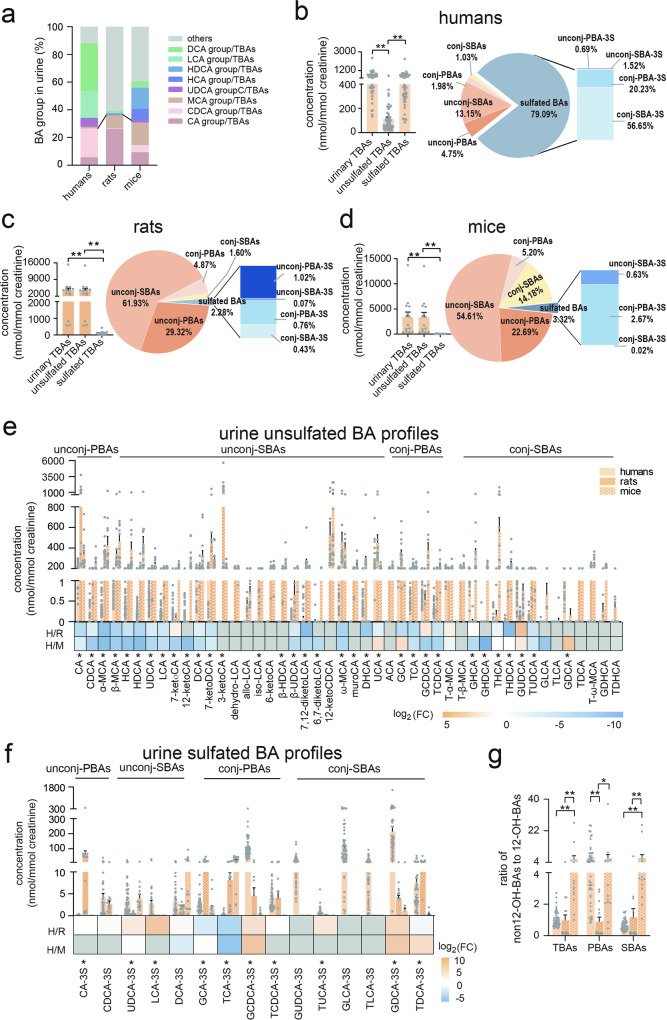
Urine BAs in humans, rats, and mice. **a** The percentage of urinary BA groups among humans, rats, and mice. **b** Total BA levels and unsulfated/sulfated BA compositions in human urine. **c** Total BA levels and unsulfated/sulfated BA compositions in rat urine. **d** Total BA levels and unsulfated/sulfated BA compositions in mouse urine. **e** Urine unsulfated BA profiles and comparison among humans, rats, and mice. **f** Urine sulfated BA profiles and comparison among humans, rats, and mice. **g**. The ratio of urine non-12-OH-BAs to 12-OH-BAs among humans, rats, and mice. The colors blue, orange, and gray in heat maps indicate decreased, increased, and below detection limits in at least one group, respectively. Human (*n* = 50), rat (*n* = 14), and mice (*n* = 14). CA group: sum of CA, TCA, GCA, and their sulfated metabolites, CDCA group: sum of CDCA, TCDCA, GCDCA, and their sulfated metabolites, MCA group: sum of α-/β-MCA, and T-α-/β-MCA, UDCA group: sum of UDCA, TUDCA, GUDCA, and their sulfated metabolites, HCA group: sum of HCA, THCA, and GHCA, HDCA group: sum of HDCA, THDCA, and GHDCA, LCA group: sum of LCA, TLCA, GLCA, and their sulfated metabolites, DCA group: sum of DCA, TDCA, GDCA, and their sulfated metabolites, others: excluding above mentioned BA groups. Unconj-PBAs unconjugated primary BAs, unconj-SBAs unconjugated secondary BAs, conj-PBAs conjugated primary BAs, conj-SBAs conjugated secondary BAs, unconj-PBA-3S sulfated unconj-PBA at C-3, unconj-SBA-3S sulfated unconj-SBA at C-3, conj-PBA-3S sulfated PBA at C-3, conj-SBA-3S sulfated conj-SBAs at C-3, 12-OH-BAs 12-hydroxylated-BAs. TBAs total BAs, PBAs primary BAs, SBAs secondary BAs. FC fold change of humans to rats or mice, H humans, R rats, M mice, * indicates *p* < 0.05, ** indicates *p* < 0.01. Error bars depict the standard error of the mean (SEM).

Although the percentages of each BA group in TBAs showed similar trends to those in serum, the detailed BA profiles revealed marked differences between humans and each rodent species (Fig. 3a). Human urine BA profiles were dominated by sulfated BAs (in nmol/mmol creatinine), which accounted for 79.08% of the total BA profile (Fig. 3b). Total sulfated BAs comprised 20.23% conj-PBAs and 56.65% conj-SBAs, whereas only 0.69% were unconj-PBAs and 1.52% were unconj-SBAs. Meanwhile, unsulfated BAs comprised 4.75% unconj-PBAs and 13.15% unconj-SBAs, with only 1.95% and 1.03% conj-PBAs and conj-SBAs, respectively. However, sulfated BAs in rats and mice both comprised <4.00% of total urinary BAs. The unsulfated unconj-BAs (including unconj-PBAs and unconj-SBAs) dominated the urinary BAs, where there were 29.32% unconj-PBAs and 61.93% unconj-SBAs in rats (Figs. 3c) and 22.69% unconj-PBAs and 54.61% unconj-SBAs in mice (Fig. 3d).

Of the 45 detected unsulfated BAs, 36 differed significantly (*p* < 0.05) among humans, rats, and mice (Fig. 3e and Supplementary Table 3). Of these, 28 differed significantly (*p* < 0.05) between humans and rats and 26 *(p* < 0.05) differed between humans and mice (Supplementary Table 3). Most human to rat (H/R) or mouse (H/M) BA ratios were <1. Moreover, except for 3 sulfated BAs that were undetected in both rats and mice, 10 of the remaining 12 detected sulfated BAs differed significantly (*p* < 0.05) among the three groups (Fig. 3f). In summary, the top five BAs by percentage in urine were sulfated GDCA (GDCA-3S), sulfated GCDCA (GCDCA-3S), GLCA-3S, UCA, and sulfated GUDCA (GUDCA-3S) in humans; 3-ketoCA, CA, 12-ketoCDCA, UCA and ω-MCA in rats; and 12-ketoCDCA, β-MCA, HDCA, GHDCA and α-MCA in mice. The ratio of total non12-OH-BAs to the total 2-OH-BAs was highest in mice (5.83), followed by humans (1.03), and rats (0.97). Moreover, the ratio of non12-OH-PBAs to 12-OH-PBAs was lowest in rats (0.89), while it exceeded 5 in humans and mice (Fig. 3g). The ratio of non12-OH-SBAs to 12-OH-SBAs was also lowest in humans (0.65), attributed to the high percentages of the DCA group in TBAs (Fig. 3g).

### Feces bile acid profiles of humans, rats, and mice

**Fig. 4:**
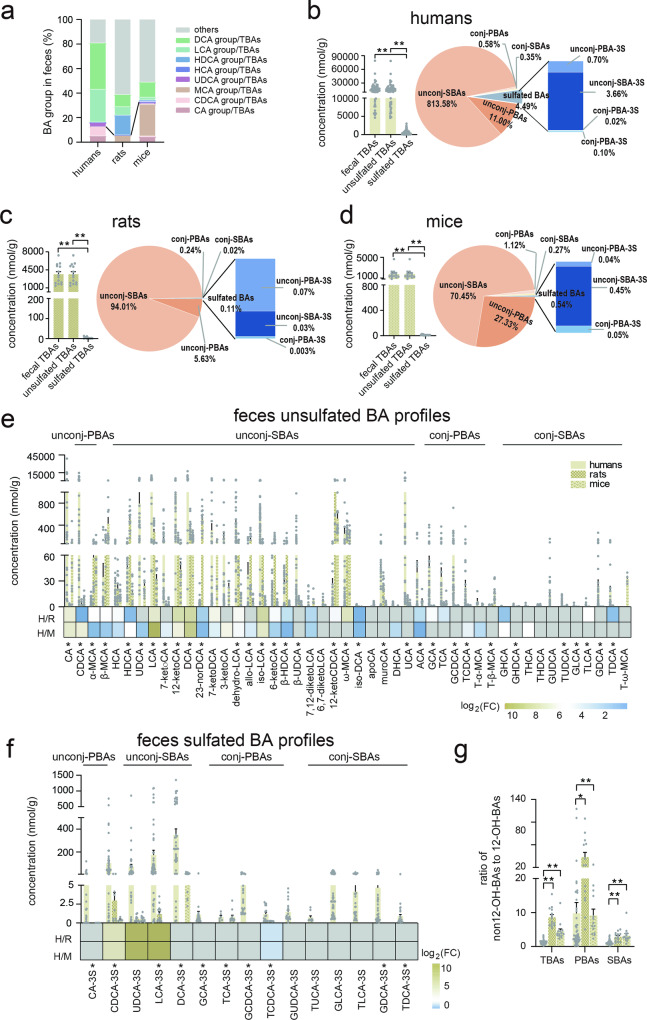
Feces BAs in humans, rats, and mice. **a** The percentage of fecal BA groups among humans, rats, and mice. **b** Total BA levels and unsulfated/sulfated BA compositions in human feces. **c** Total BA levels and unsulfated/sulfated BA compositions in rat feces. **d** Total BA levels and unsulfated/sulfated BA compositions in mouse feces. **e** Feces unsulfated BA profiles and comparison among humans, rats, and mice. **f** Feces sulfated BA profiles and comparison among humans, rats, and mice. **g** The ratio of serum non-12-OH-BAs to 12-OH-BAs among humans, rats and mice. The colors blue, tawny, and gray in heat maps indicate decreased, increased, and below detection limits in at least one group, respectively. Human (*n* = 50), rat (*n* = 14), and mice (*n* = 14). CA group: sum of CA, TCA, GCA, and their sulfated metabolites, CDCA group: sum of CDCA, TCDCA, GCDCA, and their sulfated metabolites, MCA group: sum of α-/β-MCA, and T-α-/β-MCA, UDCA group: sum of UDCA, TUDCA, GUDCA, and their sulfated metabolites, HCA group: sum of HCA, THCA, and GHCA, HDCA group: sum of HDCA, THDCA, and GHDCA, LCA group: sum of LCA, TLCA, GLCA, and their sulfated metabolites, DCA group: sum of DCA, TDCA, GDCA, and their sulfated metabolites, others: excluding above mentioned BA groups. Unconj-PBAs unconjugated primary BAs, unconj-SBAs unconjugated secondary BAs, conj-PBAs conjugated primary BAs, conj-SBAs conjugated secondary BAs, unconj-PBA-3S sulfated unconj-PBA at C-3, unconj-SBA-3S sulfated unconj-SBA at C-3, conj-PBA-3S sulfated PBA at C-3, conj-SBA-3S sulfated conj-SBA at C-3, 12-OH-BAs 12-hydroxylated-BAs. TBAs total BAs, PBAs primary BAs, SBAs secondary BAs. FC fold change of humans to rats or mice, H humans, R rats, M mice, * indicates *p* < 0.05, ** indicates *p* < 0.01. Error bars depict the standard error of the mean (SEM).

In fecal BAs, the highest percentage of PBAs was observed in mice (32.63%), followed by humans (12.67%), while rats exhibited the lowest percentage (5.61%). Regarding SBAs, both the DCA group and LCA group percentages increased across all three species, with humans showing the highest levels (Fig. 4a). Human feces had the highest total BA levels (17602.88 nmol/g), followed by rats (3644.83 nmol/g) and mice (1785.96 nmol/g). Sulfated BAs excreted into feces comprised <5% of total fecal BA levels: 4.47% in humans, 0.08% in rats, and 0.70% in mice (Fig. 4b–d). Unconjugated BAs (unconj-BAs), including PBAs and SBAs, consistently dominated in all three species. Unconj-PBAs and unconj-SBAs comprised 0.57% and 83.23% in human feces, 0.26% and 94.34% in rat feces, and 1.67% and 67.62% in mouse feces. However, 33 of the 47 detected BAs differed significantly (*p* < 0.05) among human, rat, and mouse BA profiles, whereas 28 BAs were significantly different (*p* < 0.05) between humans and rats and 29 differed significantly (*p* < 0.05) between humans and mice (Fig. 4e and Supplementary Table 4). It was noteworthy that the majority of unsulfated BAs were lower in human feces than in rats and mice (Fig. 4f), whereas most sulfated BAs were higher in humans than in rats and mice (Fig. 4f). Similarly, sulfated unconj-BAs, such as CDCA-3S, UDCA-3S, LCA-3S, and DCA-3S, were the major components in human sulfated BA profiles. To summarize, the five most abundant BAs by percentages in feces were DCA, LCA, CDCA, 12-ketoLCA, and CA in humans; 12-ketoCDCA, HDCA, DCA, ω-MCA and LCA in rats; and 12-ketoCDCA, β-MCA, ω-MCA, DCA and CA in mice. Additionally, the lowest ratio of total non12-OH-BAs to total 12-OH-BAs was observed in humans at 1.22, while in rats and mice, the ratio was more than three times higher (Fig. 4g). Consistent with the findings in serum and urine, the ratios of non12-OH-SBAs to 12-OH-SBAs were lowest in humans (Fig. 4g).

### Sulfated BAs and gut microbial transformed BAs

**Fig. 5:**
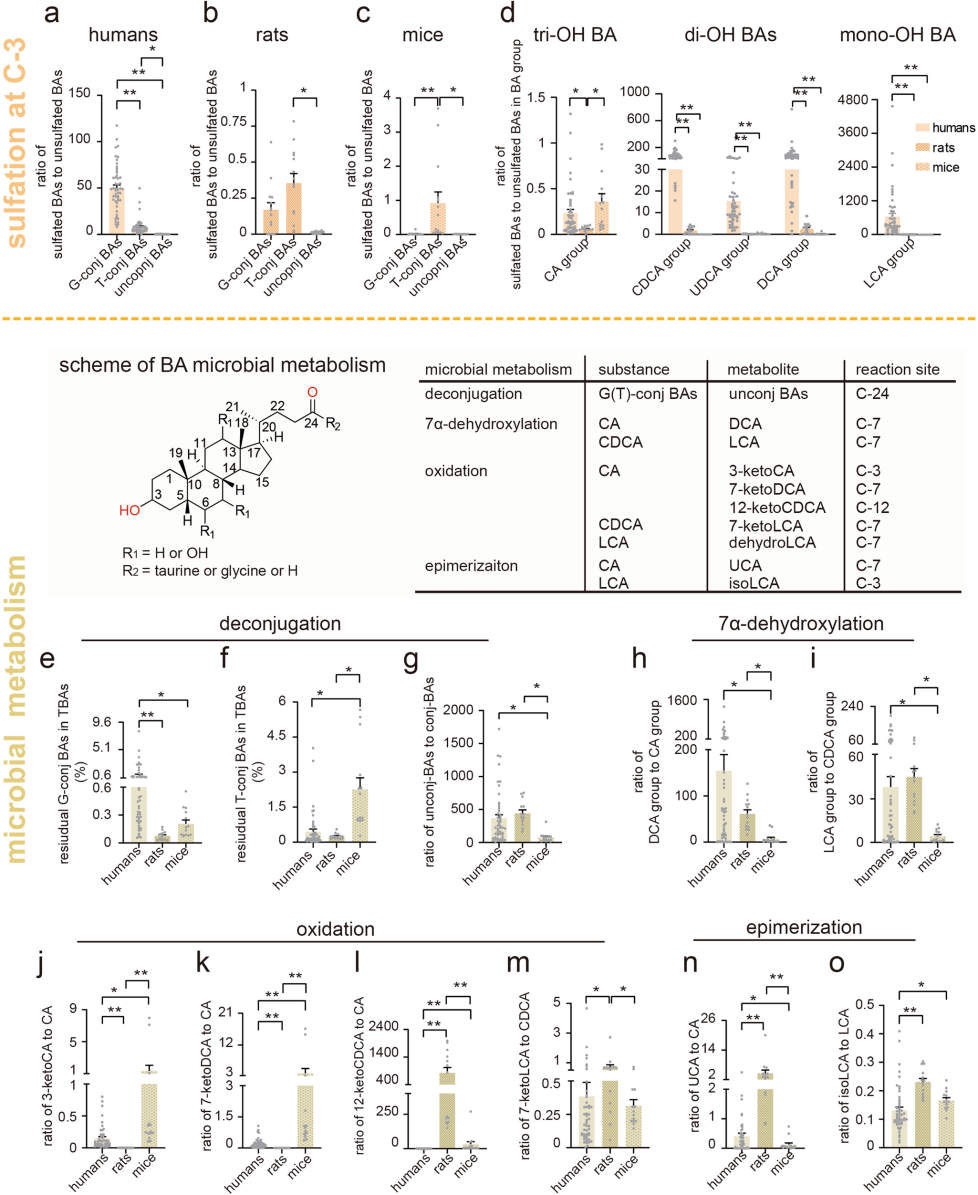
BA indices (percentages or ratios) of sulfated BAs and gut microbial transformed BAs. **a**–**c** The ratio of sulfated BAs to unsulfated BAs in humans, rats, and mice. **d** The ratio of sulfated BAs to unsulfated BAs in BA group among humans, rats, and mice. **e**, **f** The residual T-conj-BAs and G-conj-BAs among humans, rats, and mice. **g**–**o** The BA ratios of microbial transformed BAs. The ratio of unconj-BAs to conj-BAs (**g**), DCA group to CA group (**h**), LCA group to CDCA group (**i**), 3-ketoCA to CA (**j**), 7-ketoCA to CA (**k**), 12-ketoCDCA to CA (**l**), 7-ketoLCA to CDCA (**m**), UCA to CA (**n**) and isoLCA to CA (**o**) among humans, rats, and mice. Human (*n* = 50), rat (*n* = 14), and mice (*n* = 14). G-conj-BAs glycine-conjugated BAs, T-conj-BAs taurine-conjugated BAs, unconj-BAs unconjugated BAs, tri-OH BA trihydroxy BA, di-OH BAs dihydroxy BA, mono-OH BA monohydroxy BA. CA group: the sum of CA, TCA, and GCA. DCA group: the sum of DCA, TDCA, and GDCA. CDCA group: the sum of CDCA, TCDCA, and GCDCA. LCA group: LCA, TLCA and GLCA. * indicates *p* < 0.05, ** indicates *p* < 0.01. Error bars depict the standard error of the mean (SEM).

In the sulfation procedure of BA Phase II metabolism, C-3 is one of the potential sites for sulfate conjugation at the hydroxyl groups, Fig. 3b–d showed that conj-BA-3S (sulfated at C-3, including conj-PBA-3S and conj-SBA-3S) dominated the urinary sulfated BAs in all three species. Humans exhibited a significantly higher ratio of sulfated BAs to unsulfated BAs compared to the two rodents (Fig. 5a–c). Within humans, the highest ratio was observed for glycine-conjugated BAs (G-conj-BAs, 49.76), followed by taurine-conjugated BAs (T-conj-BAs, 8.02), and unconj-BAs (0.20) (Fig. 5a). Unlike humans, in two rodents, the highest ratio of sulfated BAs to unsulfated BAs was found in T-conj-BAs (rats: 0.44, mice: 0.56), followed by G-conj-BAs (0.17 and 0.36), and unconj-BAs (0.01 and 0.01) (Fig. 5b, c). This tendency was also observed in serum (Supplementary Fig 1a–c). Notably, the ratio of the sulfated LCA group (mono-OH) to unsulfated LCA group was the highest, while the ratio of sulfated CA group to CA group (tri-OH) was lowest in human urine (Fig. 5d and Supplementary Fig 1e). However, in rat urine, the ratio of the sulfated BAs to unsulfated BAs in DCA group (di-OH) was the highest, while the ratio of the LCA group (mono-OH) was the lowest (Fig. 5d and Supplementary Fig 1f). In mice urine, the CA group had the highest ratio, while the LCA group the lowest ratio (Fig. 5d and Supplementary Fig 1g). Furthermore, the ratio of sulfated BAs to unsulfated BAs in LCA group was the highest in all three species serum, the ratio of CA group was the lowest in human and rat serum while the ratio of CDCA group (di-OH) was the lowest in mice serum (Supplementary Fig 1d, h–j).

In feces, unconj-BAs became the most abundant BA forms after deconjugation by gut microbes, constituting more than 95% of total BAs in humans, rats and mice (Fig. 4b–d, Fig. 5e, f). G-conj-BAs constituted the abundance residual conj-BAs in humans while T-conj-BAs were the predominant residual BAs in rats and mice (Fig. 5e, f). And the ratio of unconj-BAs to conj-BAs were quite similar in both humans and rats, ranging from 365.43-443.50, whereas it was only 76.14 in mice (Fig. 5g). 7α-Dehydroxylation is another important BA transformation catalyzed by gut microbiota, in which CA and CDCA convert to DCA and LCA, respectively. The ratio of the DCA group to the CA group in humans, rats, and mice was 154.56, 61.01, and 7.40, respectively (Fig. 5h). Similarly, the ratio of the LCA group to the CDCA group in humans, rats, and mice was 37.74, 44.64, and 4.19, respectively (Fig. 5i). In the oxidation transformation of BAs, CA underwent primary oxidation at the C-3 and C-7 positions to produce 3-ketoCA and 7-ketoCA, respectively, in mice. In contrast, for rats, the primary oxidation occurred at the C-12 position, mainly resulting in the formation of 12-ketoCA (Fig. 5j–l). For CDCA the primary oxidation took place at the C-7 position, leading to the formation of 7-ketoLCA in rats (Fig. 5m). Additionally, the ratio of dehydroLCA to LCA was similar in rats and mice, and higher than in humans (Supplementary Fig 2). For BA epimerization, marked differences were also observed between humans and two rodent species. The ratio of UCA to CA was lowest in mice, followed by humans and rats (Fig. 5n). Furthermore, the ratio of isoLCA to LCA significantly lower in human compared to two rodents (Fig. 5o).

### Sex-dependent BA profiles

To investigate whether BA profiles differed by sex, we compared individual BAs and their percentages in total BAs, and unconj-PBAs, unconj-SBAs, conj-PBAs, and conj-SBAs and their corresponding proportions, as well as the ratios of sulfated BAs to BAs and SBAs to PBAs between males and females.

**Fig. 6:**
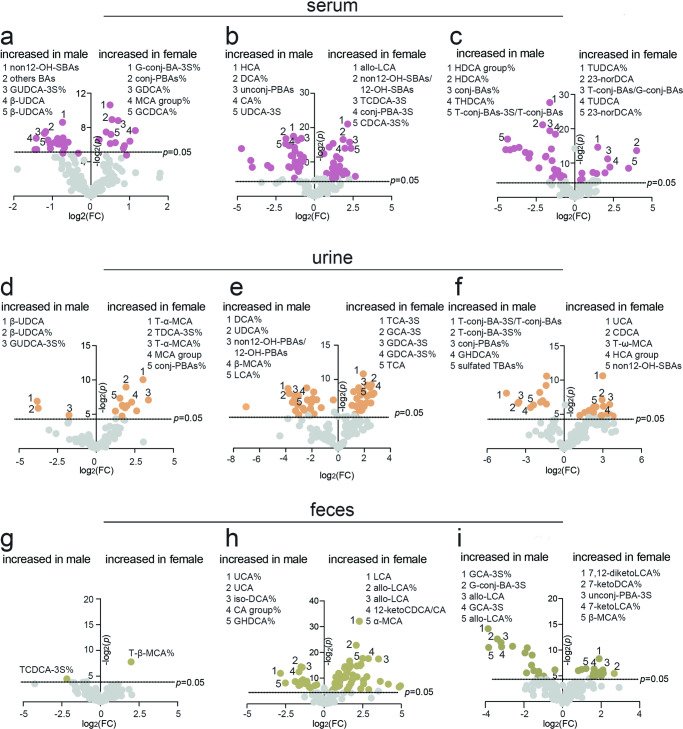
BA sex-dependent differences. **a**–**c** Human, rat, and mouse serum BA volcano plots. **d**–**f** Human, rat, and mouse urine BA volcano plots. **g**–**i** Human, rat, and mouse feces BA volcano plots. FC indicates the fold change of female to male. Human (male = 25, female = 25), rat (male = 7, female = 7), and mice (male = 7, female = 7). *p* indicates the *p* value compared with males based on the student’s *t* test. G-conj-BAs glycine-conjugated BAs, T-conj-BAs taurine-conjugated BAs, G-conj-BA-3S sulafted glycine-conjugated BAs, T-conj-BA-3S sulafted taurine-conjugated BAs, Conj-PBAs conjugated primary BAs, unconj-PBAs unconjugated primary BAs, unconj-BAs unconjugated BAs.

In serum, all variables above the dotted line (*p* = 0.05) exhibited sex-based differences (see volcano plots, Fig. 6a–c). For example, sulfated G-conj-BAs% (G-conj-BA-3S%), conj-PBAs%, GDCA%, MCA group% and GCDCA% were significantly higher (*p* < 0.05, FC (female/male) > 1.2) in human females, whereas non12-OH-SBAs, others BAs, GUDCA-3S%, β-UDCA and β-UDCA% were higher (*p* < 0.05, FC < 0.8) in human males (Supplementary Table 5). Allo-LCA, non12-OH-SBAs/12-OH-SBAs, sulfated TCDCA (TCDCA-3S), sulfated conj-PBAs (conj-PBAs-3S), and sulfated CDCA% (CDCA-3S%) were significantly higher (*p* < 0.05, FC > 1.2) in female rats, whereas HCA, DCA%, unconj-PBAs, CA% and sulfated UDCA (UDCA-3S) were significantly higher (*p* < 0.05, FC < 0.8) in male rats. In mouse serum, TUDCA%, 23-norDCA, T-conj-BAs/G-conj-BAs, TUDCA and 23-norDCA% were significantly higher (*p* < 0.05, FC > 1.2) in females, whereas HDCA group%, HDCA%, conj-BAs%, THDCA% and sulfated T-conj-BAs (T-conj-BA-3S)/T-conj-BAs were significantly higher (*p* < 0.05, FC < 0.8) in males.

In urine, T-α-MCA, sulfated TDCA% (TDCA-3S%), T-α-MCA%, MCA group and conj-PBAs%, etc., were significantly higher (*p* < 0.05, FC > 1.2) in female humans, whereas β-UDCA, β-UDCA% and GUDCA-3S were significantly higher (*p* < 0.05, FC < 0.8) in male humans (Fig. 6d and Supplementary Table 6). However, many BAs or BA proportions were higher in female and male rats. Sulfated TCA (TCA-3S), sulfated GCA (GCA-3S), GDCA-3S, GDCA-3S% and TCA, etc., were significantly higher (*p* < 0.05, FC > 1.2) in female rats, whereas DCA%, UDCA%, non12-OH-PBAs/12-OH-PBAs, β-MCA% and LCA%, etc., were significantly higher (*p* < 0.05, FC < 0.8) in male rats (Fig. 6e and Supplementary Table 6). UCA, CDCA, T-ω-MCA, HCA group and non12-OH-SBAs, etc., were significantly higher (*p* < 0.05, FC > 1.2) in female mice, whereas T-conj-BA-3S/T-conj-BAs, T-conj-BA-3S%, conj-PBAs%, GDHCA% and sulfated TBAs%, etc., were significantly higher (*p* < 0.05, FC < 0.8) in male mice (Fig. 6f and Supplementary Table 6).

In feces, only T-β-MCA% was significantly higher (*p* < 0.05, FC > 1.2) in female humans, while only TCDCA-3S was significantly higher (*p* < 0.05, FC < 0.8) in male humans (Fig. 6g and Supplementary Table 7). However, many BA levels varied between female and male rat and mice feces. LCA, allo-LCA%, allo-LCA, 12-ketoCDCA/CA and α-MCA, etc., were significantly higher (*p* < 0.05, FC > 1.2) in female rats, whereas UCA%, UCA, iso-DCA%, CA group% and GHDCA%, etc., were higher (*p* < 0.05, FC < 0.8) in male rats (Fig. 6h). Additionally, 7,12-diketoLCA%, 7-ketoLCA%, sulfated unconj-PBAs (unconj-PBA-3S), 7-ketoLCA and β-MCA%, etc., were significantly higher (*p* < 0.05, FC > 1.2) in female mice, whereas GCA-3S%, sulfated G-conj-BAs (G-conj-BA-3S), allo-LCA, GCA-3S and allo-LCA%, etc., were significantly higher (*p* < 0.05, FC < 0.8) in male mice (Fig. 6i). Collectively, marked sex-dependent differences in BA compositions were observed in human, rat, and mouse serum, urine, and feces.

## Discussion

Rats and mice are commonly used to explore the effectiveness and underlying molecular mechanisms of potential medications. Over the past two decades, several studies have shown that BAs serve as important signaling molecules in the onset and progression of some diseases. However, there are marked differences in BA metabolism and homeostasis among humans, rats, and mice, which can lead to incongruous^23^ or even contradictory results among them. Therefore, systematically comparing BA compositions and concentrations in humans and animals becomes indispensable for exploring and explaining such divergences.

The PBAs are synthesized via two major pathways: the classical and alternative pathways. The classical pathway predominantly synthesizes CA and CDCA, while the alternative pathway is also quantitatively significant and favors the synthesis of CDCA^12^. Except for CA and CDCA, rats and mice also synthesize MCA and UDCA as PBAs. The ratio of non12-OH-PBAs (CDCA group in humans, CDCA group, MCA group, and UDCA group in rats and mice) to 12-OH-PBAs (CA group) is determined by the sterol 12α-hydroxylase (CYP8B1), which has been associated with liver diseases^24,25^. Our observations indicate variations in the ratio of non-12-OH-PBAs to 12-OH-PBAs among humans, rats, and mice, as well as differences across different biological matrices. These findings may be attributed to BA metabolism and warrant further investigation for a better understanding.

BA sulfation is an important detoxification pathway, representing a distinct form of phase II metabolism for BAs in humans. The resulting metabolites are primarily excreted in urine, a fact substantiated by the presence of over four-fifths of sulfated BAs in human urine BAs and approximately a quarter in human serum^19^. However, <1% of rat and mice serum BAs and <4% of rat and mice urine BAs are sulfated. The lower sulfated BA proportions in rat and mice serum, urine, and feces indicate that sulfation is a minor pathway in BA metabolism in these rodents. This finding corroborates previous reports that rats and mice have lower BA sulfation capabilities than humans^26^. Additionally, a predominance of hepatic Sult2A1 expression and dehydroepiandrosterone sulfotransferase activity is not associated with a greater abundance of bile acid sulfates in mice^27^. Moreover, in healthy human urine, 82.04% of sulfated BAs are conj-BAs, which is consistent with previous reports^19^ and suggests that BA sulfation after amidation with glycine or taurine are the major ways to excrete excess BAs from the human body. Despite rats and mice have lower BA sulfation capabilities, the extent of sulfation in conj-BAs is much higher than unconj-BAs. Our findings support previous reports suggesting that the extent of BA sulfation is inversely proportional to the number of hydroxy groups in humans^14^. However, this pattern is not entirely consistent in our results for rats and mice. Among the three di-OH BA groups, the ratios of the sulfated BAs to unsulfated BAs have no marked difference in both human and rat urine, but not in mouse urine. Additional studies are therefore required to confirm and better understand these results.

Because of the higher affinity MRP3^28,29^ and higher efficiency organic anion transporting polypeptide transporter^29^ in rats than in humans, we observed that serum BA concentrations were 5.89-fold higher in rats than in humans. Additionally, the percentage of unconj-BAs in rat serum was 93.91%, which is consistent with reports of 87% unconj-BAs in rat plasma^30,31^ but significantly different from that in humans where the percentage is 35.59%. However, the percentage of conjugated to unconj-BAs in human serum was comparable to the percentage in mice, which is 41.25%^32^. Moreover, CA, CDCA, α-MCA, β-MCA, and HDCA were the dominant unconj-BAs in rat serum, which is also consistent with unconj-BAs comprising the majority of rat serum BAs^32^. Multiple hydroxy BAs in the serum correspond to urinary BA compositions, where the most abundant fraction in both rats and mice comprised unconjugated BAs and highly hydrophilic BAs, such as UCA, CA, HDCA, and β-MCA. In contrast to rats and mice, conjugated BAs represented the predominant fraction in human urine, where unsulfated conjugated BAs and sulfated conjugated BAs comprised almost 80% of total BAs. Additionally, BAs amidate mainly with glycine (58.36% in serum, 72.09% in urine) and minorly with taurine (7.14% serum and 4.79% in urine) in humans but predominantly with taurine (59.87% in serum and 6.86% in urine) in mice. However, conjugated BAs were relatively scarce (28.00% in serum and 7.65% in urine) in rats, implying that BA synthetase, particularly amidation with glycine or taurine, is distinctly different between humans and the two rodent species.

Deconjugation, which involves the removal of glycine or taurine moieties from BAs, is considered an essential function of the gut microbiome. It is often referred to as the ‘gateway reaction’ for subsequent modifications^33^. Fecal BAs in all three species consistently comprised >95% unconjugated BAs, mainly consisting of unconjugated secondary BAs. Similarly, 7α-dehydroxylation leads to a significant increase in the hydrophobic BA pool of humans, with the ratio of the DCA group to the CA group and the LCA group to the CDCA group consistently exceeding. This study highlights species differences in microbial transformation capacity among the three species, showing that the capabilities of deconjugation and 7α-dehydroxylation in humans are comparable to those in rats but much stronger than those in mice. On the contrary, humans exhibit lower capabilities of oxidation and epimerization compared to rats or mice. Additionally, LCA is mainly excreted in human feces, whereas HDCA and β-MCA are mainly excreted in rat and mice feces, respectively. These differential metabolic products reflect the differences in the compositions of gut microbiota between humans and the two rodent species.

BA profiles from serum, urine, and feces not only highlight significant differences between species but also reveal marked sex-dependent differences within each species. Our results indicate significant sex differences in the serum, urine, and feces of rats and mice. However, for humans, significant sex differences are observed in serum and urine but not in feces. This partly explains why discrepancies exist between male and female rats and mice when evaluating the effects of certain medications; in most situations, only one sex is used per study^34–37^. Here, we systematically compared sex-based differences in serum, urine, and feces from healthy humans, rats, and mice to identify variations in physiology by sex so that future studies could use greater caution when selecting a sex for a given experiment.

Comprehensive BA profiles of human, rat, and mouse serum, urine, and feces facilitate improved understanding of interspecies differences in various physiological conditions. Understanding such differences could explain discrepancies in certain experimental outcomes where humans and murine rodents are compared. Meanwhile, these differences highlight the need for researchers to exercise caution when extrapolating translational findings from murine rodents to humans.

## Methods

### Materials

Sixty-five bile acid standards with a purity of >98% were purchased from Steraloids or Sigma. BA abbreviations are summarized in Supplementary Table 1.

### Sample collection

Humans: All ethical regulations relevant to human research participants were followed. All human studies were approved by the Ethics Committee of The Sixth People’s Hospital Affiliated with Shanghai Jiaotong University School of Medicine (ethics approval number: 2021-YS-084) in accordance with the World Medical Association’s Declaration of Helsinki. Fifty age- and sex-matched healthy volunteers (25 female, average age 25.6 ± 4.5; 25 male, average age 26.1 ± 5.2) were recruited under signed informed consent for this study. Serum and urine samples were collected the morning after fasting, whereas fecal samples were collected at the first defecation after overnight fasting. All collected samples were stored at -80°C until they could be analyzed.

Rats and mice: We have complied with all relevant ethical regulations for animal use. Fourteen sex-matched Sprague Dawley rats (8-week, 200-260 g) and fourteen sex-matched C57BJ/6L mice (8-week, 18–20 g) were used in this study. Serum samples were collected from the eye frames, urine and feces were individually collected in metabolic cages after fasting 12 h. All studies were approved by The Sixth People’s Hospital affiliated to Shanghai Jiaotong University School of Medicine (ethics approval number: NO.2021-0110).

### Blank matrix preparation

Human serum, urine, and feces samples were used to prepare the blank matrix of serum, urine, and feces respectively based on referenced reports^38,39^. 150 mg of activated coal was added to 1 mL of serum or urine. The mixture was vortexed for 2 h, followed by centrifugation at 4°C and 13,000 rpm for 15 min. The resulting supernatant was filtered through a 0.22 μm filter membrane to obtain the blank matrix. For feces samples, ~5 mg of freeze-dried fecal matter was extracted with 200 μL of methanol and homogenized for 6 min, followed by centrifugation at 13000 rpm and 4°C for 15 min. The supernatant was diluted 50 folds with methanol. 150 mg of activated coal was added to 1 mL of the diluted solution. The mixture was vortexed for 2 h. Subsequently, centrifugation was performed at 13,000 rpm and 4°C for 15 min. The resulting supernatant was filtered through a 0.22 μm filter membrane to obtain the blank matrix.

### Preparation of calibration curve and internal standard solutions

Each BA standard was prepared in methanol at a final concentration of 1 mmol/L as stock solutions. These stock solutions were mixed and serially diluted with methanol to generate 11 levels in the calibration curve ranging from 1 to 2000 nmol/L. 50 μL of each concentration point of the calibration curve was evaporated to dryness, and the resulting dried standards were stored at −80 °C. Prior to sample preparation, the dried standards were reconstituted with blank matrix. Each internal standard (IS), including cholic acid-d_4_ (CA-d_4_), usrodeoxycholic acid-d_4_ (UDCA-d_4_), lithocholic acid-d_4_ (LCA-d_4_), glycocholic acid-d_4_ (GCA-d_4_), glycodeoxycholic acid-d_4_ (GDCA-d_4_), glycochenodeoxycholic acid-d_4_ (GCDCA-d_4_), hyocholic acid-d_5_ (HCA-d_5_), hyodeoxycholic acid-d_5_ (HDCA-d_5_), deoxycholic acid-3-sulfate-d_4_ (DCA-3S-d_4_), and glycocholic acid-3-sulfate-d_4_ (GCA-3S-d_4_) was weighed and dissolved in methanol to obtain stock solutions (1 mmol/L). The stock solutions of IS were further diluted and mixed in methanol to obtain a working solution containing each IS at 50 nmol/L.

### Sample preparation

A 50 µL aliquot of serum or urine sample was extracted with 200 µL working solution containing 10 IS (50 nmol/L). The extract was allowed to stand at −20°C for 30 min, then centrifuged at 13,000 rpm for 15 min. The resulting supernatant was transferred to another tube and vacuum-dried. The residual was reconstituted in 25 µL methanol and 25 µL water, and the resulting supernatant was used for quantification analysis. For the feces sample, ~5 mg freeze-dried fecal matter was extracted with 200 µL methanol, and the resultant supernatant was subjected to 50- and 5-fold dilution with methanol for human feces and rat/mouse feces, respectively. Next, a 50 µL dilution aliquot was combined with 100 µL working solution containing 10 IS (50 nmol/L), vortexed for 5 min, and centrifuged at 13,000 rpm for 15 min. The supernatant was transferred into a sample tube for quantification analysis.

### UPLC-TQMS analysis

All samples were quantitatively measured using UPLC-TQMS (Waters Corp., Milford, MA). Separations were performed on a Waters CORTECS C18 column, 100 mm × 2.1 mm, 1.7 µm (Waters, Milford, MA) at 45 °C. Mobile phase A comprised 5 mM ammonium acetate water with 0.01% acetate, and mobile phase B comprised LC-MS grade acetonitrile and methanol (9:1 v/v) with a flow rate of 0.4 mL/min. The mobile phase linear gradient was initiated at 80% phase A, 0–1 min 73% A, held 73% A from 1 to 2 min, decreased to 65% A from 2.0 to 3.5 min, then 40% A at 8.5 min, 0% A at 9.5 min and held to 11 min, increased back to 80% A at 11.1 min and balanced until 13 min. The injection volume for all tested samples was 5 µL. The mass spectrometer was run on negative mode at a source temperature of 150 °C and a desolvation gas temperature of 500 °C. Data acquisition was performed using MassLynx version 4.1. All parameters of detected BAs are summarized in Supplementary Table 8.

### Sample quantification

BA concentration was calculated by calibration curve. The calibration curve was established with the abscissa and the ordinate, which was the peak area ratio and the mass concentration ratio of the target BA to be measured and the corresponding IS substance, respectively. In particular, urinary BA concentration was normalized to creatinine concentration and expressed as nmol/mmol creatinine, while fecal BA concentration was normalized to freeze-dried feces weight and expressed as nmol/g.

### LC-MS method validation

Linear regressions were constructed using 1/x weighted least-squares linear regression, plotting the peak area ratios of the analyte to the IS. The lowest concentrations on the calibration curve, determined based on signal-to-noise ratios of three and ten, were defined as the limit of detection and the limit of quantitation, respectively. The detailed information in serum, urine, and feces was listed in Supplementary Tables 9–11, respectively. The accuracy was evaluated by recovery. Three levels of calibrators, STD-L (10 nmol/L), STD-M (100 mmol/L), and STD-H (1000 nmol/L) were evaporated, and then redissolved in the blank matrix, respectively. All the BAs were tested with six replicates in each level and the recovery was calculated as the ratio of the measured concentration to the theoretical concentration. The result of accuracy was listed in Supplementary Table 12. The precision of the assay was also assessed at three levels with three replicates respectively. The relative standard deviation of the results within 1 day was used as an indicator for evaluating intra-day precision, while measurements were taken on three consecutive days for inter-day precision. The result of intra-day precision was listed in Supplementary Table 13, and the result of inter-day precision was summarized in Supplementary Table 14.

### Statistics and reproducibility

All data were analyzed in GraphPad Prism 9 (GraphPad Software, USA). The data were expressed as mean ± SEM. Kruskal–Wallis test, followed by a post-hoc Dunn’s multiple-comparison, was used to evaluate the significance of differences. *p* < 0.05 were considered statistically significant, and the criteria for different metabolites between males and females were a *p* value of < 0.05 and a fold change (FC) of >1.2 or <0.8.

### Reporting summary

Further information on research design is available in the Nature Portfolio Reporting Summary linked to this article.

### Supplementary information

supplementary information

supplementary data 1

reporting summary

## Data Availability

The source data behind the graphs in the figures can be found in Supplementary Data 1. Raw data of BA quantification is publicly available at Metabolomics Workbench. All other data are available from the corresponding author upon reasonable request.

## Code availability

The analysis methods in this article are all conventional, and no new methods or algorithms were generated.
