# Supplementary material for: Comparative profiling of serum, urine, and feces bile acids in humans, rats, and mice
Source: Commun Biol. 2024 May 27;7:641. doi: 10.1038/s42003-024-06321-3 (PMC11130135; doi:10.1038/s42003-024-06321-3)
Supplement: Supplementary file 1 — supplementary information [file 42003_2024_6321_MOESM1_ESM.pdf]

Comparative Profiling of Serum, Urine, and Feces Bile Acids in Humans, Rats, and Mice

Dan Zheng[1], Kun Ge[1], Chun Qu[1], Tao Sun[1], Jieyi Wang[1], Wei Jia[2], Aihua Zhao[1]*

[1] Center for Translational Medicine, Shanghai Key Laboratory of Diabetes Mellitus and Shanghai Key Laboratory of Sleep Disordered Breathing, Shanghai Sixth People's Hospital Affiliated to Shanghai Jiao Tong University School of Medicine, Shanghai 200233, China

[2] Department of Pharmacology and Pharmacy, University of Hong Kong, Hong Kong, China

Correspondence

Aihua Zhao, Center for Translational Medicine, Shanghai Key Laboratory of Diabetes Mellitus and Shanghai Key Laboratory of Sleep Disordered Breathing, Shanghai Sixth People's Hospital Affiliated to Shanghai Jiao Tong University School of Medicine, Shanghai 200233, China

Email: zhah@sjtu.edu.cn

The following Supplementary Material is available for this paper.

1. Supplementary Tables 1-14 (available in this file)

2. Supplementary Figures 1-2 (available in this file)

Supplementary Table 1 Bile acids abbreviation

abbreviation	full name	abbreviation	full name
CA	cholic acid	GCDCA	glycochenodeoxycholic acid
CDCA	chenodeoxycholic acid	TCDCA	taurochendeoxycholic acid
α-MCA	α-muricholic acid	T-α-MCA	tauro α-muricholic acid
β-MCA	β-muricholic acid	T-β-MCA	tauro β-muricholic acid
HCA	λ-muricholic acid	GHCA	glyco λ-muricholic acid
HDCA	hyodeoxycholic acid	GHDCA	glycohyodeoxycholic acid
UDCA	ursodeoxycholic acid	THCA	tauro λ-muricholic acid
LCA	lithocholic acid	THDCA	taurohyodeoxycholic acid
7-ketoLCA	7-keto lithocholic acid	GUDCA	glycoursodeoxycholic acid
12-ketoLCA	12-keto lithocholic acid	TUDCA	tauroursodeoxycholic acid
DCA	deoxycholic acid	GLCA	glycolithocholic acid
23-norDCA	23-nordeoxycholic acid	TLCA	taurolithocholic acid
7-ketoDCA	7-keto deoxycholic acid	GDCA	glycodeoxycholic acid
3-ketoDCA	3-keto deoxycholic acid	TDCA	taurodeoxycholic acid
dehydro-LCA	dehydro-lithocholic acid	T-ω-MCA	tauro ω-muricholic acid
allo-LCA	allolithocholic acid	GDHCA	glycodehydrocholic acid
iso-LCA	isolithocholic acid	TDHCA	taurodehydrocholic acid
6-ketoLCA	6-keto lithocholic acid	CA-3S	cholic acid 3-sulfate
β-HDCA	3β-hyodeoxycholic acid	CDCA-3S	chenodeoxycholic acid 3-sulfate
β-UDCA	3β-ursodeoxycholic acid	UDCA-3S	ursodeoxycholic acid 3-sulfate
7,12-diketoLCA	7,12-diketo lithocholic acid	LCA-3S	lithocholic acid 3-sulfate
6,7-diketoLCA	6,7-diketo lithocholic acid	DCA-3S	deoxycholic acid 3-sulfate
12-ketoCDCA	12-keto chenodeoxycholic acid	GCA-3S	glycocholic acid 3-sulfate
ω-MCA	ω-muricholic acid	TCA-3S	taurocholic acid 3-sulfate
β-CA	3β-cholic acid	GCDCA-3S	glycochenodeoxycholic acid 3-sulfate
iso-DCA	isodeoxycholic acid	TCDCA-3S	taurochendeoxycholic acid 3-sulfate
apoCA	apocholic acid	GUDCA-3S	glycoursodeoxycholic acid 3-sulfate
muroCA	murocholic acid	TUDCA-3S	tauroursodeoxycholic acid 3-sulfate
DHCA	dehydrocholic acid	GLCA-3S	glycolithocholic acid 3-sulfate
UCA	ursocholic acid	TLCA-3S	taurolithocholic acid 3-sulfate
ACA	allocholic acid	GDCA-3S	glycodeoxycholic acid 3-sulfate
GCA	glycocholic acid	TDCA-3S	taurodeoxycholic acid 3-sulfate
TCA	taurocholic acid		

Supplementary Table 2 Serum bile acid concentrations in humans, rats and mice (nmol/L)

	humans	rats	mice	humans vs rats (*p*)	humans vs mice (*p*)	rats vs mice (*p*)	humans vs rats vs mice (*p*)
CA	117.71±111.46	5488.67±1945.20	274.26±202.19	< 0.01	< 0.01	< 0.01	< 0.01
CDCA	390.60±328.28	2159.39±1251.77	78.77±28.14	< 0.01	< 0.01	< 0.01	< 0.01
α-MCA	7.22±1.67	1747.96±1019.35	21.26±12.90	< 0.01	< 0.01	< 0.01	< 0.01
β-MCA	N.D.	1271.33±742.23	317.56±178.88			< 0.01	< 0.01
HCA	16.72±13.82	147.17±72.42	22.07±35.31	< 0.01	> 0.05	< 0.01	< 0.01
HDCA	8.19±7.09	2841.21±1478.57	41.55±25.29	< 0.01	< 0.01	< 0.01	< 0.01
UDCA	137.36±123.42	297.29±191.36	70.18±36.02	< 0.01	< 0.05	< 0.01	< 0.01
LCA	20.06±13.97	22.72±16.24	14.39±5.65	> 0.05	> 0.05	> 0.05	> 0.05
7-ketoLCA	7.92±12.54	86.90±78.76	5.85±3.33	< 0.01	> 0.05	< 0.01	< 0.01
12-ketoLCA	14.44±11.4	6.48±1.10	7.04±1.83	< 0.01	< 0.01	< 0.05	< 0.01
DCA	438.56±334.85	136.47±82.23	336.19±95.51	< 0.01	> 0.05	< 0.01	< 0.01
23-norDCA	3.94±2.09	1.41±0.26	59.17±63.01	< 0.01	< 0.01	< 0.01	< 0.01
7-ketoDCA	N.D.	212.94±228.96	74.00±67.41			> 0.05	> 0.05
3-ketoCA	7.07±2.06	208.72±107.75	8.95±4.72	< 0.01	< 0.01	< 0.01	< 0.01
allo-LCA	4.46±4.20	4.10±3.10	2.39±1.28	< 0.01	> 0.05	< 0.01	< 0.05
iso-LCA	37.22±39.41	2.45±0.89	2.39±1.28	< 0.01	< 0.01	> 0.05	< 0.01
6-ketoLCA	3.34±0.76	74.09±57.37	3.17±1.67	< 0.01	> 0.05	< 0.01	< 0.01
β-HDCA	5.04±3.87	89.16±44.47	2.80±1.15	< 0.01	< 0.01	< 0.01	< 0.01
β-UDCA	315.01±390.47	4.02±3.52	3.62±1.84	< 0.01	< 0.01	< 0.05	< 0.01
6,7-diketoLCA	N.D.	35.23±22.80	9.50±3.80			< 0.01	< 0.01
12-ketoCDCA	N.D.	9978.67±8373.49	635.67±405.15			< 0.01	< 0.01
ω-MCA	4.91±3.86	263.19±140.31	168.88±72.23	< 0.01	< 0.01	< 0.05	< 0.01
muroCA	N.D.	54.17±39.7	10.72±6.83			< 0.01	< 0.01
UCA	N.D.	87.28±92.97	9.51±9.81			< 0.05	< 0.05
ACA	N.D.	279.24±113.85	20.72±12.82			< 0.01	< 0.01
GCA	221.95±207.89	71.87±31.25	6.34±3.8	< 0.01	< 0.01	< 0.01	< 0.01
TCA	28.16±27.07	484.76±169.27	778.05±550.64	< 0.01	< 0.01	> 0.05	< 0.01
GCDCA	975.02±947.00	6.28±4.27	1.15±0.19	< 0.01	< 0.01	< 0.01	< 0.01
TCDCA	86.41±77.00	76.98±49.97	22.04±19.75	> 0.05	< 0.01	< 0.01	< 0.01
T-α-MCA	10.66±4.15	263.12±109.57	192.27±168.07	< 0.01	< 0.01	< 0.05	< 0.01
T-β-MCA	N.D.	71.93±33.55	482.68±402.9	< 0.01	< 0.01	< 0.01	< 0.01
GHCA	19.95±16.09	1.29±0.29	1.08±0.15	< 0.01	> 0.05	> 0.05	> 0.05
GHDCA	8.22±9.33	32.21±29.39	N.D.	< 0.01			< 0.01
THCA	7.32±2.95	N.D.	7.36±0.81			> 0.05	> 0.05
THDCA	1.77±0.74	199.36±118.71	51.20±26.63	< 0.01	< 0.01	< 0.01	< 0.01
GUDCA	118.91±154.42	1.32±0.25	1.68±0.60	< 0.01	< 0.01	> 0.05	< 0.01
TUDCA	5.28±6.91	6.28±2.77	54.76±50.07	> 0.05	< 0.01	< 0.05	< 0.01
GLCA	11.15±8.34	N.D.	N.D.				
TLCA	2.05±1.82	1.47±0.30	2.40±0.55	> 0.05	> 0.05	> 0.05	> 0.05
GDCA	315.32±276.02	1.74±1.75	0.44±0.28	< 0.01	< 0.01	< 0.01	< 0.01
TDCA	35.20±24.46	9.02±4.18	105.40±38.64	< 0.01	< 0.01	< 0.01	< 0.01
T-ω-MCA	27.70±4.16	21.95±5.27	2330.46±2066.88	> 0.05	< 0.05	< 0.01	< 0.01
CA-3S	N.D.	1.73±0.46	N.D.				
CDCA-3S	3.59±3.89	2.07±1.03	N.D.	< 0.05			< 0.01
UDCA-3S	3.14±2.80	4.14±2.76	1.16±0.42	< 0.05	< 0.01	< 0.01	< 0.01
LCA-3S	20.87±28.21	2.81±1.21	N.D.	< 0.01	< 0.01	< 0.01	< 0.01
DCA-3S	3.17 ± 2.29	N.D.	N.D.				
GCA-3S	2.49 ± 1.74	N.D.	N.D.				
TCA-3S	1.23 ± 0.49	1.45 ± 0.29	18.55 ± 16.77	> 0.05	< 0.01	< 0.01	< 0.01
GCDCA-3S	206.66 ± 116.17	2.20 ± 0.76	N.D.	< 0.01			< 0.01
TCDCA-3S	15.29 ± 8.58	10.39 ± 7.96	N.D.	< 0.05			> 0.05
GUDCA-3S	75.16 ± 407.74	N.D.	N.D.				
TUDCA-3S	3.60 ± 4.04	1.12 ± 0.11	1.03 ± 0.03	< 0.01	< 0.01	< 0.01	< 0.01
GLCA-3S	509.08 ± 468.34	N.D.	N.D.				
TLCA-3S	90.32 ± 84.24	6.48 ± 5.22	1.74 ± 0.06	< 0.01	< 0.01	< 0.01	< 0.01
GDCA-3S	267.72± 239.83	1.44 ± 0.35	N.D.	< 0.01			< 0.01
TDCA-3S	14.16 ± 15.90	2.72 ± 1.48	1.68 ± 0.43	< 0.01	< 0.05	> 0.05	< 0.01

N.D. means no detected in samples, *p* means *p* value

Supplementary Table 3 Urine bile acid concentrations in humans, rats and mice (nmol/mmol creatinine)

	humans	rats	mice	humans vs rats (*p*)	humans vs mice (*p*)	rats vs mice (*p*)	humans vs rats vs mice (*p*)
CA	22.94±27.36	1061.37±1001.52	140.67±151.42	< 0.01	< 0.01	< 0.01	< 0.01
CDCA	0.32±0.64	1.96±1.84	54.27±63.5	< 0.01	< 0.01	< 0.01	< 0.01
α-MCA	0.36±0.28	151.76±131.48	227.57±323.69	< 0.01	< 0.01	> 0.05	< 0.01
β-MCA	1.64±2.09	143.93±188.82	265.04±289.12	< 0.01	< 0.01	> 0.05	< 0.01
HCA	0.5±0.63	9.85±10.26	186.41±272.47	< 0.01	< 0.01	< 0.01	< 0.01
HDCA	0.38±0.47	24.59±37.5	238.67±274.55	< 0.01	< 0.01	< 0.01	< 0.01
UDCA	0.65±0.54	4.99±7.71	42.92±57.04	< 0.01	< 0.01	< 0.01	< 0.01
LCA	0.28±0.26	3.87±5.81	8.81±7.35	< 0.01	< 0.01	< 0.05	< 0.01
7-ketoLCA	0.16±0.26	0.1±0.33	20.95±20.85	< 0.01	< 0.01	< 0.01	< 0.01
12-ketoLCA	0.18±0.34	N.D.	83.66±110.19		< 0.01		< 0.01
DCA	3.72±5.79	21.72±34.41	127.74±179.56	< 0.01	< 0.01	< 0.05	< 0.01
7-ketoDCA	11.93±15.49	246.53±417.01	98.94±88.84	< 0.01	< 0.01	< 0.01	< 0.01
3-ketoCA	N.D.	1397.75±1520.97	23.11±24.97			< 0.01	< 0.05
dehydro-LCA	N.D.	6.49±7.29	8.9±8.46			> 0.05	> 0.05
allo-LCA	N.D.	0.43±0.56	0.67±0.68			> 0.05	> 0.05
iso-LCA	N.D.	0.98±1.46	1.73±1.6	< 0.01	< 0.01	< 0.05	< 0.01
6-ketoLCA	N.D.	12.06±16.8	17.85±19.2			> 0.05	> 0.05
β-HDCA	N.D.	4.17±6.24	37.89±69.11			< 0.01	< 0.01
β-UDCA	0.08±0.12	0.65±1.38	3.92±5.96	< 0.05	< 0.01	< 0.01	< 0.01
7,12-diketoLCA	0.1±0.23	64.97±99.3	6.03±9.4	< 0.01	< 0.01	< 0.05	< 0.01
6,7-diketoLCA	0.03±0.14	N.D.	1.41±3.43		< 0.05		< 0.05
12-ketoCDCA	N.D.	322.75±480.76	627.52±881.19			> 0.05	> 0.05
ω-MCA	2.78±3.49	261.3±341.42	186.83±239.3	< 0.01	< 0.01	> 0.05	< 0.01
muroCA	N.D.	2.6±3.07	32.08±47.4			< 0.01	< 0.01
DHCA	0.37±0.42	76.57±158.82	N.D.	< 0.01			< 0.01
UCA	49.16±88.57	252.01±290.7	16.51±16.68	< 0.01	< 0.05	< 0.01	< 0.01
ACA	N.D.	2.19±3.91	15.86±19.66			< 0.05	< 0.05
GCA	3.53±2.45	144.48±140.85	1.08±1.54	< 0.01	< 0.01	< 0.01	< 0.01
TCA	0.64±0.67	31.25±25.98	13.66±21.2	< 0.01	< 0.01	> 0.05	< 0.01
GCDCA	1.23±0.92	0.53±0.5	175.48±283.24	< 0.05	> 0.01	< 0.01	< 0.01
TCDCA	0.35±0.32	4.8±4.53	27.29±50.69	< 0.05	< 0.05	> 0.05	< 0.01
T-α-MCA	5.11±7.42	2.91±3.15	12.56±25.28	> 0.05	> 0.05	> 0.05	> 0.05
T-ß-MCA	N.D.	9.64±8.99	27.28±43.73			> 0.05	< 0.05
GHCA	2.52±2.84	0.03±0.1	136.65±214.75	< 0.01	< 0.01	< 0.01	< 0.01
GHDCA	0.01±0.07	N.D.	15.05±23.83	< 0.01			< 0.01
THCA	0.05±0.13	0.16±0.42	356.83±528.23	< 0.01	< 0.01	< 0.01	< 0.01
THDCA	0.01±0.03	4.71±5.31	48.38±82.29	< 0.01	< 0.01	< 0.05	< 0.01
GUDCA	1.55±1.66	0.15±0.09	0.74±0.65	< 0.01	> 0.05	< 0.05	< 0.01
TUDCA	0.11±0.27	1.64±2.75	1.42±1.35	< 0.01	< 0.01	> 0.05	< 0.01
GLCA	N.D.	N.D.	0.65±1.05				
TLCA	N.D.	N.D.	0.04±0.16				
GDCA	1.29±1.5	N.D.	0.16±0.59	< 0.01	< 0.05	> 0.05	< 0.01
TDCA	N.D.	1.43±2.21	1.16±1.83			> 0.05	> 0.05
T-ω-MCA	N.D.	27.39±15.04	43.02±57.58			> 0.05	> 0.05
GDHCA	N.D.	0.3±0.56	20.23±11.67			< 0.01	< 0.01
TDHCA	N.D.	0.35±0.5	N.D.				
CA-3S	0.05±0.09	61.15±94.02	N.D.	< 0.01			< 0.01
CDCA-3S	4.18±6.56	2.59±2.95	N.D.	> 0.05			> 0.05
UDCA-3S	3.53±4.14	0.46±0.27	4.21±3.71	< 0.01	> 0.05	< 0.01	< 0.01
LCA-3S	2.76±5.36	0.01±0.04	N.D.	< 0.01			< 0.01
DCA-3S	3.28±3.83	1.86±1.57	17.36±26.03	> 0.05	> 0.05	> 0.05	> 0.05
GCA-3S	2.57±2.19	14.62±14.34	1.74±2.45	> 0.05	> 0.05	< 0.05	< 0.05
TCA-3S	0.22±0.31	8.17±6.15	28.2±16.66	< 0.01	< 0.01	> 0.05	< 0.01
GCDCA-3S	102.93±64.1	4.52±6.95	0.31±0.58	< 0.01	< 0.01	> 0.05	< 0.01
TCDCA-3S	3.92±2.84	4.16±5.43	N.D.	> 0.05			< 0.01
GUDCA-3S	24.23±27.47	N.D.	N.D.				
TUDCA-3S	0.84±0.98	0.07±0.1	N.D.	> 0.05			< 0.01
GLCA-3S	109.6±110.79	N.D.	N.D.				
TLCA-3S	17.96±19.44	N.D.	N.D.				
GDCA-3S	213.64±241.85	3.74±3.39	1.14±2.72	< 0.01	< 0.01	> 0.05	< 0.01
TDCA-3S	7.23±9.78	13.08±13.86	0.25±0.56	> 0.05	< 0.01	< 0.01	< 0.01

N.D. means no detected in samples, *p* means *p* value.

Supplementary Table 4 Feces bile acid concentrations in humans, rats and mice (nmol/g)

	humans	rats	mice	humans vs rats (*p*)	humans vs mice (*p*)	rats vs mice (*p*)	humans vs rats vs mice (*p*)
CA	2710.62±7440.24	1.91±0.91	93.23±118.72	< 0.01	< 0.01	< 0.01	< 0.01
CDCA	1469.81±2799.69	3.42±2.7	9.88±5.42	< 0.01	< 0.01	> 0.05	< 0.01
α-MCA	6.75±4.14	57.42±45.54	70.48±49.12	< 0.01	< 0.01	> 0.05	< 0.01
β-MCA	34.57±86.54	135.51±80.76	445.67±420.92	< 0.01	< 0.01	> 0.05	< 0.01
HCA	27.75±34.31	21.61±17.18	12.94±5.35	> 0.05	> 0.05	> 0.05	> 0.05
HDCA	45.57±28.9	499.82±200.91	16.7±10.82	< 0.01	< 0.01	< 0.01	< 0.01
UDCA	792.87±1258.38	N.D.	24.8±11.96		< 0.01		< 0.01
LCA	3643.61±2385.74	272.69±189.41	30.95±21.04	< 0.01	< 0.01	< 0.01	< 0.01
7-ketoLCA	143.45±185.93	3±1.94	4.41±3.19	< 0.01	< 0.01	> 0.05	< 0.01
12-ketoLCA	968.81±892.44	135.96±53.54	43.08±31.85	< 0.01	< 0.01	> 0.05	< 0.01
DCA	5298.34±3224.7	344.52±173.89	169.15±72.14	< 0.01	< 0.01	< 0.01	< 0.01
23-norDCA	5.62±5.09	N.D.	N.D.				< 0.01
7-ketoDCA	304.79±851.6	N.D.	118.29±126.73		> 0.05		> 0.05
3-ketoCA	171.23±484.22	N.D.	23.47±15.47		> 0.05		> 0.05
dehydro-LCA	402.77±600.14	85.32±61.87	12.23±11.18	< 0.01	< 0.01	< 0.01	< 0.01
allo-LCA	37.6±62.36	128.02±122.44	4.82±5.23	< 0.01	> 0.05	< 0.01	< 0.01
iso-LCA	480.8±455.9	61.17±45.26	5.04±3.57	< 0.01	< 0.01	< 0.01	< 0.01
6-ketoLCA	18.93±16.57	139.3±57.61	5.52±4.16	< 0.01	< 0.01	< 0.01	< 0.01
β-HDCA	27.16±22.93	112.02±60.04	4.09±2.23	< 0.01	< 0.01	< 0.01	< 0.01
β-UDCA	104.89±186.86	6.5±2.69	2.98±1.69	< 0.01	< 0.01	< 0.01	< 0.01
7,12-diketoLCA	4.52±6.37	N.D.	7.9±5.43		> 0.05		> 0.05
6,7-diketoLCA	N.D.	6.98±4.86	1.83±0.5			> 0.05	> 0.05
12-ketoCDCA	25.86±32.7	1295.32±1020.37	528.82±394.92	< 0.01	< 0.01	< 0.05	< 0.01
ω-MCA	244.56±662.57	296.37±156.74	198.98±104.89	< 0.01	< 0.05	> 0.05	< 0.01
iso-DCA	1.84±0.83	0.3±0.03	0.27±0.04	< 0.05	< 0.01	> 0.05	< 0.01
apoCA	N.D.	N.D.	3.28±2.08				
muroCA	41.96±43.9	23.04±10.05	3.09±1.71	> 0.05	< 0.05	< 0.05	< 0.05
DHCA	2.88±1.73	N.D.	N.D.				
UCA	1125.5±2859.62	6.67±3.8	3.08±1.87	< 0.01	< 0.01	< 0.01	< 0.01
ACA	40.01±69.32	N.D.	6.99±6.34		< 0.01		< 0.01
GCA	46.98±84.63	0.79±0.26	0.76±0.36	< 0.01	< 0.01	> 0.05	< 0.01
TCA	34.93±72.95	3.37±1.24	4.93±4.35	> 0.05	> 0.05	> 0.05	> 0.05
GCDCA	65.7±117.03	0.99±0.33	1.12±0.49	< 0.01	< 0.01	> 0.05	< 0.01
TCDCA	22.81±56.59	1.4±0.83	0.79±0.48	< 0.01	< 0.01	< 0.05	< 0.01
T-α-MCA	6.69±6.49	N.D.	2.76±1			> 0.05	> 0.05
T-β-MCA	1.31±0.7	2.34±0.31	16.75±9.94	> 0.05	< 0.01	> 0.05	< 0.01
GHCA	0.75±0.27	N.D.	N.D.				
GHDCA	1.18±1.4	0.3±0.1	0.62±0	< 0.05	> 0.05	> 0.05	< 0.05
THCA	0.73±0.8	N.D.	N.D.				
THDCA	1.21±0.95	1.67±0.72	N.D.	> 0.05			> 0.05
GUDCA	12.71±26.48	N.D.	N.D.				
TUDCA	2.77±3.66	0.47±0.12	1.08±0.65	< 0.05	> 0.05	> 0.05	< 0.05
GLCA	0.64±0.73	0.09±0.05	0.05±0.02	< 0.01	< 0.01	> 0.05	< 0.01
TLCA	2.4±2.05	0.33±0.11	N.D.	< 0.01	< 0.01	> 0.05	< 0.01
GDCA	24.55±47.3	0.31±0.06	0.24±0.01	< 0.01	< 0.01	> 0.05	< 0.01
TDCA	17.96±31.46	1.42±0.12	N.D.	< 0.01	< 0.01	> 0.05	< 0.01
T-ω-MCA	N.D.	N.D.	32.64±5.77				
CA-3S	15.51±25.67	2.99±2.9	N.D.	< 0.01			< 0.01
CDCA-3S	102.88±155.75	N.D.	N.D.	< 0.01	< 0.01		< 0.01
UDCA-3S	70.3±143.21	0.52±0.39	0.53±0.38	< 0.01	< 0.01	> 0.05	> 0.05
LCA-3S	174.03±282.05	1.19±1.02	N.D.	< 0.01			< 0.01
DCA-3S	349.41±339.97	N.D.	9.13±6.39		< 0.01		< 0.01
GCA-3S	1.09±2.22	N.D.	1±0.91	< 0.01			< 0.01
TCA-3S	0.77±0.44	N.D.	0.71±0.73	< 0.01	< 0.01	< 0.01	< 0.01
GCDCA-3S	5.04±5.3	N.D.	N.D.	< 0.01			< 0.01
TCDCA-3S	1.19±0.95	0.23±0.03	N.D.	< 0.01			< 0.01
GUDCA-3S	1.46±1.25	N.D.	N.D.				
TUDCA-3S	0.72±0.64	N.D.	N.D.	> 0.05			
GLCA-3S	7.71±10.4	N.D.	N.D.	< 0.01			
TLCA-3S	4.16±4.97	N.D.	N.D.	< 0.01			
GDCA-3S	4.67±8.14	N.D.	0.43±0.19		< 0.01		< 0.01

N.D. means no detected in samples, *p* means *p* value.

Supplementary Table 5 Sex dependent differential BAs of serum in humans, rats and mice

	Humans			rats			mice	
	FC	*p*		FC	*p*		FC	*p*
G-conj-BA-3S%	1.41	0.0006	allo-LCA	4.44	4.67E-07	allo-LCA	4.44	4.67E-07
conj-PBA%	1.46	0.0021	HCA group%	0.39	5.43E-06	HCAgroup%	0.39	5.43E-06
GDCA%	1.63	0.0022	HCA%	0.39	5.66E-06	HCA%	0.39	5.66E-06
non12-OH-SBAs	0.60	0.0026	unconj-PBA%	0.56	8.41E-06	unconj-PBAs%	0.56	8.41E-06
MCAgroup/%	2.22	0.0050	DCA%	0.28	8.77E-06	DCA%	0.28	8.77E-06
12-OHBAs%	1.31	0.0052	non12-OH-SBAs/12-OH-SBAs	3.62	1.05E-05	non12-OH-SBAs/12-OH-SBAs	3.62	1.05E-05
conj-SBAs%	1.32	0.0056	CA%	0.47	1.24E-05	CA%	0.47	1.24E-05
GUDCA-3S%	0.43	0.0063	TCDCA-3S	4.85	1.81E-05	TCDCA-3S	4.85	1.81E-05
GCDCA%	1.43	0.0068	12-ketoCDCA%	2.30	2.34E-05	12-ketoCDCA%	2.30	2.34E-05
UDCA%	0.61	0.0090	UDCA-3S	0.28	2.52E-05	UDCA-3S	0.28	2.52E-05
β-UDCA	0.37	0.0093	DCA group%	0.32	3.6E-05	DCA group%	0.32	3.6E-05
Others BAs	0.55	0.0095	12-OH-BAs/non12-OH-BAs	0.41	5.11E-05	Others BAs%	1.94	4.91E-05
β-UDCA%	0.46	0.0108	conj-PBA-3S	3.93	6.17E-05	12-OH-BAs/non12-OHBAs	0.41	5.11E-05
T-α-MCA%	2.01	0.0118	UCA%	0.04	6.65E-05	conj-PBA-3S	3.93	6.17E-05
unconj-SBA	0.66	0.0129	unconj-SBA%	1.53	0.000188	UCA%	0.04	6.65E-05
UDCA	0.52	0.0131	CA group%	0.53	0.000224	unconj-SBA%	1.53	0.000188
UDCA group%	0.61	0.0132	DCA	0.35	0.000277	CA group%	0.53	0.000224
GCA%	1.55	0.0132	DCA group	0.38	0.000304	DCA%	0.35	0.000277
T-conj-BAs%	1.42	0.0143	non12-OH-BAs/12-OH-BAs	2.69	0.000328	non12-OH-BAs/12-OH-BAs	2.69	0.000328
CDCA	0.55	0.0148	6,7-diketo LCA%	3.15	0.000533	DCA group	0.39	0.000374
CDCA%	0.57	0.0149	CA	0.57	0.000633	CA-group	0.61	0.000381
UDCA-3S	0.48	0.0153	UCA	0.06	0.000727	6,7-diketoLCA%	3.15	0.000533
MCA group	1.82	0.0170	MCA group%	0.53	0.000792	CA	0.57	0.000633
unconj-PBAs%	0.62	0.0197	12-OH-BAs	0.60	0.000851	UCA%	0.06	0.000727
TCA%	1.79	0.0208	UDCAgroup%	0.42	0.000972	MCA group%	0.53	0.000792
UDCA-3S%	0.48	0.0213	T-conj-BA-3S/T-conj-BAs	2.35	0.001187	12-OH-BAs	0.60	0.000851
GUDCA-3S	0.37	0.0232	UDCA%	0.42	0.001335	UDCA group%	0.42	0.000972
unconj-PBA	0.61	0.0273	α-MCA%	0.44	0.001564	T-conj-BA-3S/T-conj-BAs	2.35	0.001187
unconj-SBA%	0.80	0.0311	7-ketoDCA%	0.12	0.00196	LCA group	3.11	0.001317
HCA%	0.60	0.0312	LCA	3.10	0.002324	UDCA%	0.42	0.001335
GCDCA-3S	0.71	0.0353	muroCA%	0.32	0.002604	α-MCA%	0.44	0.001564
BA-3S/BAs in UDCA group	0.70	0.0358	7-ketoLCA%	2.62	0.002624	7-ketoDCA%	0.12	0.00196
unconj-BA-3S/conj-BAs	1.90	0.0362	unconj-SBA-3S	0.52	0.002642	LCA%	3.10	0.002324
DCA group%	1.32	0.0398	TCA-3S%	0.06	0.002896	muroCA%	0.32	0.002604
iso-LCA	0.52	0.0432	UDCA-3S%	0.34	0.003064	7-ketoLCA%	2.62	0.002624
non12-OH-BAs/12-OH-BAs	0.54	0.0433	7-ketoDCA	0.14	0.003217	unconj-SBA-3S	0.52	0.002642
conj-PBA-3S	0.73	0.0452	β-MCA%	0.46	0.003325	TCA-3S	0.06	0.002896
TCA	1.73	0.0466	Sulfated TBAs	2.24	0.003445	UDCA-3S	0.34	0.003064
			HCA	0.49	0.00397	7-ketoDCA	0.14	0.003217
			HCA group	0.49	0.00404	β-MCA	0.46	0.003325
			LCA group%	2.60	0.004652	Sulfated TBAs	2.24	0.003445
			LCA%	2.55	0.0048	HCA	0.49	0.00397
			BA-3S/BAs in CDCA group	3.91	0.00615	HCA group	0.49	0.00404
			unconj-SBA-3S%	0.49	0.006599	LCA group%	2.60	0.004652
			TLCA-3S	3.27	0.006929	LCA	2.55	0.0048
			conj-PBA-3S%	3.41	0.008463	BA-3S/BAs in CDCA group	3.91	0.00615
			BA-3S/BAs in DCA group	3.52	0.008477	unconj-SBA-3S%	0.49	0.006599
			6,7-diketoLCA	2.90	0.008781	TLCA-3S	3.27	0.006929
			TCDCA-3S%	4.30	0.009511	conj-PBA-3S%	3.41	0.008463
			T-α-MCA	1.72	0.009778	BA-3S/BAs in DCA group	3.52	0.008477
			THDCA	2.20	0.011318	6,7-diketoLCA	2.90	0.008781
			7-ketoLCA	3.57	0.013013	TCDCA-3S	4.30	0.009511
			12-ketoCDCA	3.13	0.01402	T-α-MCA	1.72	0.009778
			conj-SBA-3S	2.45	0.016054	THDCA	2.20	0.011318
			CDCA-3S	2.47	0.016944	7-ketoLCA	3.57	0.013013
			unconj-PBA-3S	6.25	0.018133	12-ketoCDCA	3.13	0.01402
			TCDCA	2.26	0.018671	conj-SBA-3S	2.45	0.016054
			Others BAs	2.61	0.022018	CDCA-3S	2.47	0.016944
			non12-OHSBAs	2.26	0.026615	unconj-PBA-3S	6.25	0.018133
			GCDCA%	1.56	0.027579	TCDCA	2.26	0.018671
			TLCA-3S%	2.85	0.028907	others BAS	2.61	0.022018
			TUDCA-3S	1.14	0.029746	muroCA	0.41	0.025181
			unconj-PBA-3S%	4.75	0.029831	non12-OH-SBAs	2.26	0.026615
			TUDCA%	0.24	0.034752	GCDCA%	1.56	0.027579
			SBAs	2.08	0.037406	TLCA-3S%	2.85	0.028907
			GCDCA	2.14	0.038892	TUDCA-3S	1.14	0.029746
			unsulfated TBAs%	1.00	0.04315	unconj-PBA-3S%	4.75	0.029831
			sulfatedTBA%	1.94	0.04315	T-ω-MCA	1.35	0.031622
			unconj-SBAs	2.02	0.044671	TUDCA	0.24	0.034752
			UDCA group	0.50	0.048303	SBAs	2.08	0.037406
						GCDCA	2.14	0.038892
						unsulfated TBAs%	1.00	0.04315
						Sulfated TBAs%	1.94	0.04315
						unconj-SBAs	2.02	0.044671
						UDCA group	0.50	0.044793

FC means fold change of female to male. *p* means *p* value.

Supplementary Table6 Sex dependent differential BAs of urine in humans, rats and mice

humans			rats			mice		
	FC	*p*		FC	*p*		FC	*p*
conj-PBAs	2.86	0.00095	TCA-3S	3.79	0.000555	UCA	7.95	0.000634
TDCA-3S%	1.95	0.001992	GCA-3S	5.85	0.001682	GDHCA	0.39	0.000654
conj-PBAs%	1.70	0.006154	GDCA-3S	4.66	0.001715	GUDCA%	0.38	0.001685
T-α-MCA	3.23	0.0074	GDCA-3S%	5.49	0.002158	dehydro-LCA%	0.27	0.003409
β-UDCA	0.27	0.008315	unconj-BA-3S/conj-BAs	3.00	0.002259	T-conj-BA-3S/T-conj-BAs	0.04	0.003671
MCA group	2.22	0.009145	ω-MCA%	0.19	0.002295	CDCA	7.69	0.007118
LCA%	1.80	0.012576	DCA%	0.07	0.002438	CA group%	0.31	0.007821
DCA-3S%	2.00	0.013234	CA group%	2.29	0.003315	conj-PBA-3S%	0.09	0.008976
conj-PBAs	1.82	0.013925	TCA	3.76	0.003437	BA-3S/BAs in CA group	0.28	0.009095
DHCA%	1.91	0.016255	CDCA%	0.27	0.003454	TCA-3S%	0.08	0.010071
β-UDCA%	0.27	0.016922	GCA-3S%	6.68	0.003929	T-conj-BA-3S%	0.08	0.010152
TDCA-3S	2.48	0.022206	unconj-PBA-3S%	3.27	0.003991	CA%	0.38	0.010986
α-MCA%	1.54	0.022725	UDCA%	0.08	0.004034	T-ω-MCA	11.58	0.011494
			G-conj-BA-3S%	6.23	0.004475	unsulfated TBAs	1.05	0.012085
			G-conj-BA-3S	5.52	0.004484	sulfated TBAs%	0.19	0.012085
			CA-3S%	3.30	0.004763	HCA group	8.23	0.013826
			β-MCA%	0.12	0.005034	non12-OH-SBAs	5.56	0.014615
			non12-OH-PBAs/12-OH-PBAs	0.10	0.005137	non12-OH-BAs	5.34	0.014987
			BA-3S/BAs in CDCA group	4.69	0.005274	SBAs	4.90	0.015021
			ACA%	0.00	0.006333	GDHCA%	0.16	0.015649
			CDCA-3S%	2.71	0.006584	unsulfated TBAs	4.61	0.017781
			HCA group%	0.26	0.006748	urinary TBAs	4.49	0.018628
			DCA group%	0.32	0.007577	7-ketoLCA	3.79	0.021223
			others BAs%	0.78	0.007683	non12-OH-PBAs	4.85	0.025038
			dehydro-LCA%	0.20	0.007726	UDCA-3S	3.02	0.025741
			HCA%	0.27	0.007922	T-conj-BAs	10.38	0.028839
			LCA%	0.10	0.008001	unconj-SBAs	4.67	0.0300522
			LCA group%	0.10	0.008074	DCA group	7.66	0.0338043
			non12-OH-BAs/12-OH-BAs	0.09	0.009819	TCA	12.34	0.0338094
			BA-3S/BAs in DCA group	4.51	0.010359	CDCA%	2.33	0.0346074
			UDCA group%	0.24	0.010465	DCA group	7.34	0.0347166
			DHCA%	5.68	0.010474	DCA	7.57	0.0348411
			conj-PBAs	3.38	0.010829	HDCA group	4.93	0.0350674
			12-ketoCDCA%	0.12	0.012802	T-β-MCA	13.96	0.0370057
			CA%	2.09	0.013261	HCA	8.20	0.0391593
			3-ketoCA%	2.72	0.013814	PBAs	3.73	0.0418762
			TCA%	2.60	0.014615	UDCA group	4.86	0.0428461
			GCA	3.95	0.01489	UDCA	5.67	0.0431313
			G-conj-BA-3S	3.91	0.014965	THCA	10.56	0.0453252
			β-UDCA%	0.01	0.015423	others BAs	4.22	0.0481542
			MCA group%	0.41	0.016835	12-ketoCDCA	6.18	0.0496888
			UDCA-3S%	0.16	0.017811	unconj-SBAs	4.67	0.0300522
			UCA%	0.23	0.018495	DCA group	7.66	0.0338043
			CA group	3.08	0.020119	TCA	12.34	0.0338094
			GCA%	3.54	0.021184	CDCA%	2.33	0.0346074
			6-ketoLCA%	0.17	0.021598	DCA group	7.34	0.0347166
			G-conj-BAs	3.35	0.022591			
			iso-LCA%	0.11	0.022957			
			unconj-SBAs%	0.82	0.026328			
			muroCA%	0.28	0.031752			
			conj-PBAs%	2.47	0.032791			
			HDCA%	0.12	0.034013			
			HDCAgroup%	0.21	0.037065			
			GUDCA%	0.23	0.038446			
			PBAs	2.37	0.038521			
			unsulfated TBAs%	0.99	0.040457			
			sulfated TBAs%	1.61	0.040457			
			unconj-PBAs%	1.36	0.04267			
			CA	2.88	0.049482			

FC means fold change of female to male. *p* means *p* value.

Supplementary Table7 Sex dependent differential BAs of feces in humans, rats and mice

humans			rats			mice		
	FC	*p*		FC	*p*		FC	*p*
T-β-MCA	0.41	2.17E-05	LCA	4.86	2.11E-10	GCA-3S%	0.07	5.44E-05
TCDCA-3S%	0.22	0.044852	LCAgroup	4.87	2.18E-10	G-conj-BA-3S	0.10	0.000219
			allo-LCA%	4.23	1.37E-07	allo-LCA	0.11	0.00035
			α-MCA	5.64	4.79E-06	unconj-SBA-3S%	0.17	0.000582
			12-ketoCDCA/CA	7.32	5.14E-06	GCA-3S	0.11	0.000622
			allo-LCA	11.53	5.59E-06	allo-LCA%	0.07	0.000686
			TCDCA	3.14	9.76E-06	Residual G-conj-BAs%	0.28	0.000721
			non12-OH-SBAs	3.17	2.2E-05	conj-PBA-3S	0.26	0.001418
			HCA	5.22	2.51E-05	TCDCA%	0.34	0.001911
			12-ketoCDCA	5.02	3.38E-05	7,12-diketoLCA%	3.72	0.003124
			non12-OH-PBAs	3.24	3.45E-05	LCA%	0.33	0.007386
			PBAs	3.09	3.46E-05	LCA group%	0.33	0.007517
			iso-LCA	4.34	3.73E-05	β-MCA%	2.32	0.011745
			MCA group	3.20	4.17E-05	7-ketoLCA%	3.11	0.012308
			TBAs	2.54	4.46E-05	MCA group%	1.98	0.013377
			Unsulfated TBAs	2.54	4.6E-05	unconj-PBAs%	0.76	0.014043
			unconj-PBAs	3.17	4.61E-05	unconj-PBA-3S	4.26	0.014215
			non12-OH-BAs	2.65	4.64E-05	iso-LCA%	0.36	0.015735
			UCA	0.35	4.68E-05	apoCA	0.42	0.017324
			non12-OH-BAs	2.63	5.14E-05	β-MCA	3.86	0.018383
			isoDCA%	0.37	5.21E-05	MCA group	3.26	0.02083
			SBAs	2.51	6.34E-05	LCA group/CDCA group	0.27	0.020888
			unconj-SBAs	2.51	6.42E-05	3-ketoCA%	4.33	0.022022
			othersBAs%	2.96	8.51E-05	non12-OH-PBAs	3.05	0.022407
			CA group%	0.32	0.000178	7-keto DCA%	6.41	0.022859
			GLCA	2.78	0.00021	GCDCA%	0.43	0.027442
			LCA%	1.93	0.000265	unconj-PBAs%	3.22	0.031285
			UCA%	0.14	0.000266			
			LCA group	1.93	0.000267			
			dehydro-LCA	3.63	0.000414			
			12-ketoCDCA%	1.91	0.000488			
			α-MCA%	2.17	0.000578			
			unconj-SBA-3S	6.81	0.000667			
			non12-OH-PBAs/12-OH-PBAs	4.39	0.0007			
			iso-LCA%	1.69	0.000735			
			β-MCA	2.66	0.000759			
			DCA group/CA group	2.39	0.000837			
			G-conj-BAs%	0.39	0.001546			
			Residual G-conj-BAs%	0.39	0.001546			
			CDCA group	3.54	0.001645			
			LCA-3S	5.84	0.00176			
			non12-OH-SBAs/12-OH-SBAs	1.96	0.001885			
			β-UDCA%	0.36	0.002239			
			HDCA group%	0.46	0.002308			
			sulfated TBAs	12.41	0.002332			
			β-UDCA	1.84	0.002356			
			HDCA%	0.46	0.00236			
			CA%	0.27	0.00242			
			GCA%	0.35	0.002451			
			LCA group/CDCA group	2.02	0.002526			
			GCDCA%	0.40	0.002572			
			GHDCA%	0.18	0.00364			
			T-conj-BAs	1.62	0.003871			
			UDCA group	3.35	0.003898			
			12-ketoLCA	1.74	0.004071			
			muroCA%	0.45	0.00459			
			unconj-PBAs-3S	17.89	0.004959			
			TCA%	0.35	0.005516			
			β-UDCA%	0.72	0.005727			
			unconj-BAs/conj-BAs	1.78	0.006805			
			6-ketoLCA%	0.32	0.007011			
			DCA	1.99	0.007131			
			DCA group	2.00	0.007152			
			HCA%	1.93	0.007262			
			12-ketoLCA%	0.66	0.00738			
			HCA group%	1.90	0.007718			
			THDCA	1.78	0.008051			
			unconj-BA-3S-conj-BAs	1.74	0.0083			
			12-OH-BAs	1.90	0.008962			
			CDCA	3.07	0.009027			
			G-conj-BA-3S	29.03	0.010683			
			unconj-SBA%	0.52	0.011127			
			T-conj BAs/G-conj BAs	1.62	0.012772			
			con-BAs%	0.54	0.013366			
			unconj-BAs%	1.00	0.014103			
			sulfated TBAs%	5.18	0.017407			
			unsulfated TBAs%	1.00	0.017407			
			conj-SBAs%	7.44	0.021346			
			unconj-PBA-3S%	2.84	0.021709			
			LCA-3S%	2.84	0.021709			
			non12-OH-BAs/12-OH-BAs	1.47	0.023081			
			CDCA-3S	11.48	0.032267			
			conj-PBAs	1.33	0.038717			
			others BAs%	1.14	0.043552			

FC means fold change of female to male. *p* means *p* value.

Supplementary Table 8 Parameters of detected bile acids

compound	parent	daughter	Cone (V)	Collision (V)	IS
dehydro-LCA	373.3	373.3	44	5	LCA-d_4
allo-LCA/iso-LCA/LCA	375.3	375.3	55	15	LCA-d_4
23-norDCA	377.3	377.3	56	5	UDCA-d_4
6-ketoLCA/7-ketoLCA/12-ketoLCA/apoCA	389.3	389.3	55	10	LCA-d_4/LCA-d_4/LCA-d_4/CA-d_4
muroCA/β-UDCA/β-HDCA/UDCA/HDCA/CDCA/DCA/iso-DCA	391.3	391.3	60	10	UDCA-d_4/UDCA-d_4/HDCA-d_5/UDCA-d_4/HDCA-d_5/UDCA-d_4/UDCA-d_4/UDCA-d_4
DHCA	401.3	401.3	40	10	HCA-d_5
7,12-diketoLCA/6,7-diketoLCA	403.3	403.3	60	10	LCA-d_4/LCA-d_4
7-ketoDCA/12-ketoCDCA/3-ketoCA	405.3	405.3	65	5	UDCA-d_4/CA-d_4/CA-d_4
UCA/ω-MCA/β-CA/α-MCA/β-MCA/HCA/ACA/CA	407.3	407.3	62	10	CA-d_4/CA-d_4/CA-d_4/CA-d_4/CA-d_4/HCA-d_5/HCA-d_5/CA-d_4
GLCA	432.4	73.9	58	34	LCA-d_4
GUDCA/GHDCA/GCDCA/GDCA	448.4	73.9	58	40	GCDCA-d_4/HDCA-d_5/GCDCA-d_4/GDCA-d_4
GDHCA	458.4	73.9	50	40	GCA-d_4
GHCA/GCA	464.3	73.9	60	48	HCA-d5/GCA-d_4
TLCA	482.4	79.9	88	72	LCA-d_4
TUDCA/THDCA/TCDCA/TDCA	498.4	79.9	60	75	GCDCA-d_4/HDCA-d_5/GCDCA-d_4/GCA-d_4
TDHCA	508.4	79.9	80	75	GCA-d_4
T-ω-MCA/T-α-MCA/T-β-MCA/THCA/TCA	514.4	79.9	80	75	GCA-d_4/GCA-d_4/GCA-d_4/GCA-d_4
CA-3S	487.4	97.0	30	35	DCA-3S-d_4
LCA-3S	455.4	97.0	40	40	DCA-3S-d_4
UDCA-3S/CDCA-3S/DCA-3S	471.4	97.0	50	40	DCA-3S-d_4/DCA-3S-d_4/DCA-3S-d_4
GCA-3S	544.4	464.4	40	30	GCA-3S-d_4
TCA-3S	594.3	514.3	30	20	GCA-3S-d_4
GLCA-3S	512.4	432.4	40	30	GCA-3S-d_4
TLCA-3S	562.4	482.4	25	25	GCA-3S-d_4
GUDCA-3S/GCDCA-3S/GDCA-3S	528.4	448.4	45	30	GCA-3S-d_4/GCA-3S-d_4/GCA-3S-d_4
TUDCA-3S/TCDCA-3S/TDCA-3S	578.4	498.4	30	25	GCA-3S-d_4/GCA-3S-d_4/GCA-3S-d_4
LCA-d_4	379.4	379.4	56	18	/
UDCA-d_4	395.4	395.4	40	23	/
CA-d_4	411.3	411.3	45	20	/
GCDCA-d_4/GDCA-d_4	452.4	73.9	45	40	/
GCA-d_4	468.4	73.9	58	44	/
HCA-d_5	412.5	412.5	50	10	/
HDCA-d_5	396.5	396.5	50	10	/
DCA-3S-d_4	475.2	97.9	50	40	/
GCA-3S-d_4	548.4	468.4	40	30	/

Supplementary Table 9 Linearity, limits of detection (LOD) and quantification (LOQ) results in serum

	LOD (nmol/L)	LOQ (nmol/L)	Linear range (nmol/L)		
			human	rat	mouse
CA	0.5	1	5-500	50-2000	10-1000
CDCA	0.1	0.2	5-2000	50-2000	10-500
α-MCA	2	2	5-200	50-2000	5-200
β-MCA	2	2	/	50-2000	20-1000
HCA	0.5	1	1-100	5-500	1-200
HDCA	1	1	1-100	50-2000	1-100
UDCA	1	1	10-1000	20-1000	1-200
LCA	0.2	0.5	1-100	1-100	1-100
7-ketoLCA	0.5	1	1-100	10-500	1-100
12-ketoLCA	1	2	5-200	5-200	5-200
DCA	0.1	0.2	1-2000	10-500	20-1000
23-norDCA	1	2	5-200	5-200	5-200
7-ketoDCA	2	2	/	5-1000	5-500
3-ketoCA	2	2	5-200	10-1000	5-200
dehydro-LCA	0.5	1	/	/	/
allo-LCA	0.2	0.5	1-100	1-100	1-100
iso-LCA	0.2	0.5	1-200	1-100	1-100
6-ketoLCA	0.5	1	1-100	5-500	1-100
β-HDCA	1	1	1-100	10-500	1-100
β-UDCA	1	1	20-2000	1-100	1-100
7,12-diketoLCA	1	1	/	/	/
6,7-diketoLCA	1	2	/	5-200	5-200
12-ketoCDCA	2	2	/	50-2000	50-2000
ω-MCA	1	1	1-100	20-1000	10-500
β-CA	1	1	1-100	20-1000	10-500
isoDCA	0.2	0.5	/	/	/
apoCA	1	2	/	/	/
muroCA	1	1	/	1-200	1-100
DHCA	2	2	/	5-200	/
UCA	0.5	1	/	1-500	1-100
ACA	1	2	/	Tabl5-500	5-200
GCA	0.2	0.2	10-2000	10-500	1-100
TCA	1	2	5-500	50-2000	20-2000
GCDCA	0.5	1	20-2000	1-100	1-100
TCDCA	1	1	5-500	5-200	1-100
GHCA	0.5	0.5	1-100	1-100	1-100
THCA	1	2	5-200	5-200	5-200
GHDCA	0.5	1	1-100	1-100	/
THDCA	1	1	1-100	10-500	1-200
GUDCA	0.5	1	1-1000	1-100	1-100
TUDCA	1	1	1-100	1-100	1-200
GLCA	0.5	1	1-100	1-100	1-100
TLCA	0.5	1	1-100	1-100	1-100
GDCA	0.5	1	1-2000	1-100	1-100
TDCA	1	1	1-200	1-100	10-500
T-ω-MCA	10	20	20-1000	20-1000	20-2000
T-α-MCA	2	5	5-200	50-500	10-1000
T-β-MCA	5	5	5-200	10-500	20-2000
GDHCA	0.5	1	/	/	/
TDHCA	0.5	1	/	/	/
CA-3S	0.2	0.2	/	1-100	/
CDCA-3S	0.2	0.5	1-100	1-100	1-100
UDCA-3S	0.2	0.5	1-100	1-100	1-100
LCA-3S	0.5	1	1-200	1-100	1-100
DCA-3S	0.2	0.5	1-100	1-100	1-100
GCA-3S	0.2	0.5	1-100	1-100	1-100
TCA-3S	0.2	0.5	1-100	1-100	1-100
GCDCA-3S	0.5	1	10-1000	1-100	/
TCDCA-3S	0.5	0.5	1-100	1-100	/
GUDCA-3S	0.5	1	5-1000	/	/
TUDCA-3S	0.2	0.5	1-100	1-100	1-100
GLCA-3S	2	2	5-2000	5-200	/
TLCA-3S	0.5	1	1-500	1-100	1-100
GDCA-3S	0.5	1	10-2000	1-100	/
TDCA-3S	0.5	0.5	1-100	1-100	1-100

Supplementary Table10 Linearity, limits of detection (LOD) and quantification (LOQ) results in urine

	LOD (nmol/L)	LOQ (nmol/L)	Linear range (nmol/L)		
			human	rat	mouse
CA	0.5	0.5	1-2000	50-2000	10-1000
CDCA	0.1	0.2	1-100	1-100	1-500
α-MCA	0.5	1	1-100	50-2000	1-2000
β-MCA	1	2	5-200	20-1000	5-2000
HCA	0.5	0.5	1-100	1-100	1-2000
HDCA	1	1	1-100	5-200	1-2000
UDCA	0.5	1	1-100	1-100	1-1000
LCA	0.2	0.5	1-100	1-100	1-100
7-ketoLCA	0.5	1	1-100	1-100	1-200
12-ketoLCA	0.5	1	1-100	/	1-1000
DCA	0.1	0.2	1-500	1-200	1-2000
23-norDCA	0.5	1	/	/	/
7-ketoDCA	1	1	1-1000	1-2000	5-1000
3-ketoCA	1	1	/	10-2000	1-500
dehydro-LCA	0.2	0.5	/	1-100	1-100
allo-LCA	0.1	0.2	/	1-100	1-100
iso-LCA	0.1	0.2	1-100	1-100	1-100
6-ketoLCA	0.5	1	/	1-100	1-200
β-HDCA	0.5	1	/	1-100	1-500
β-UDCA	0.5	0.5	1-100	1-100	1-100
7,12-diketoLCA	2	5	5-200	5-1000	5-200
6,7-diketoLCA	2	5	5-200	5-200	5-200
12-ketoCDCA	1	1	/	50-2000	20-2000
ω-MCA	0.5	1	1-100	50-2000	5-2000
β-CA	0.5	1	1-100	50-2000	5-2000
isoDCA	0.2	0.5	/	/	/
apoCA	1	2	/	/	/
muroCA	0.5	1	/	1-100	1-500
DHCA	0.5	1	1-100	5-2000	/
UCA	0.5	0.5	1-2000	50-2000	1-100
ACA	0.5	1	/	1-100	1-200
GCA	0.2	0.5	1-200	1-2000	1-100
TCA	1	2	5-200	5-1000	5-200
GCDCA	0.5	1	1-100	1-100	1-2000
TCDCA	0.5	1	1-100	1-100	1-500
GHCA	0.2	0.5	1-500	1-100	1-2000
THCA	1	2	5-200	5-200	5-200
GHDCA	0.5	1	1-100	1-100	1-2000
THDCA	1	1	1-100	1-200	1-1000
GUDCA	0.5	1	1-100	1-100	1-100
TUDCA	1	1	1-100	1-100	1-100
GLCA	0.5	0.5	1-100	/	1-100
TLCA	0.5	1	/	/	1-100
GDCA	2	2	5-200	5-200	5-200
TDCA	0.5	1	/	1-100	1-100
T-ω-MCA	10	20	/	20-1000	20-500
T-α-MCA	2	5	5-1000	5-200	5-200
T-β-MCA	2	5	/	5-500	5-500
GDHCA	0.5	1	/	1-100	1-100
TDHCA	0.2	0.5	/	1-100	/
CA-3S	0.2	0.5	1-100	1-1000	/
CDCA-3S	0.2	0.5	1-500	1-100	1-100
UDCA-3S	0.2	0.5	1-500	1-100	1-100
LCA-3S	0.2	0.5	1-500	1-100	/
DCA-3S	0.2	0.5	1-200	1-100	1-500
GCA-3S	0.2	0.5	1-200	1-500	1-100
TCA-3S	0.2	0.5	1-100	1-200	1-200
GCDCA-3S	0.2	0.5	50-2000	1-200	1-100
TCDCA-3S	0.5	1	1-500	1-200	1-100
GUDCA-3S	0.5	1	5-2000	/	/
TUDCA-3S	0.2	0.5	1-100	1-100	1-100
GLCA-3S	0.5	1	1-2000	1-100	1-100
TLCA-3S	0.5	1	1-2000	/	/
GDCA-3S	0.2	0.5	1-2000	1-100	1-100
TDCA-3S	0.5	1	1-1000	1-200	1-100

Supplementary Table 11 Linearity, limits of detection (LOD) and quantification (LOQ) results in feces

	LOD (nmol/L)	LOQ (nmol/L)	Linear range (nmol/L)		
			human	rat	mouse
CA	1	1	1-2000	1-100	1-2000
CDCA	0.5	1	1-2000	1-100	5-500
α-MCA	0.5	1	1-100	5-1000	50-2000
β-MCA	0.5	1	1-100	20-2000	50-2000
HCA	1	2	5-200	5-500	5-200
HDCA	1	2	5-200	50-2000	5-500
UDCA	1	2	5-2000	5-200	10-1000
LCA	0.2	0.5	1-2000	50-2000	5-500
7-ketoLCA	1	2	5-500	5-200	5-200
12-ketoLCA	1	2	5-1000	50-2000	5-1000
DCA	0.2	0.5	1-2000	50-2000	20-2000
23-norDCA	2	5	5-200	5-200	5-200
7-ketoDCA	2	2	5-1000	5-200	10-2000
3-ketoCA	5	5	5-200	/	5-500
dehydroLCA	0.5	1	1-500	20-2000	1-500
allo-LCA	0.2	0.5	1-200	20-2000	1-100
iso-LCA	0.2	0.5	1-2000	20-1000	1-100
6-ketoLCA	1	2	5-200	50-2000	5-200
β-HDCA	1	2	5-200	50-2000	5-200
β-UDCA	1	1	1-500	1-100	1-100
7,12-diketoLCA	1	2	5-200	/	5-200
6,7-diketoLCA	2	5	/	5-200	5-200
12-ketoCDCA	5	5	5-200	50-2000	50-2000
ω-MCA	0.5	1	1-500	50-2000	10-2000
β-CA	0.5	1	1-500	50-2000	10-2000
isoDCA	0.2	0.5	1-100	1-100	1-100
apoCA	1	2	/	/	5-200
muroCA	1	1	1-100	5-200	1-100
DHCA	1	2	5-200	5-200	5-200
UCA	2	2	5-2000	5-200	5-200
ACA	1	2	5-200	5-200	5-500
GCA	0.2	0.2	1-200	1-100	1-100
TCA	2	5	5-200	5-200	5-200
GCDCA	1	1	1-500	1-100	1-100
TCDCA	0.5	1	1-200	1-100	1-100
GHCA	0.5	2	5-200	5-200	5-200
THCA	0.5	1	1-100	1-100	1-100
GHDCA	0.5	1	1-100	1-100	1-100
THDCA	0.5	1	1-100	1-100	1-100
GUDCA	0.5	1	1-100	1-100	1-100
TUDCA	0.2	0.5	1-100	1-100	1-100
GLCA	0.5	0.1	1-100	1-100	1-100
TLCA	1	2	5-200	5-200	5-200
GDCA	1	1	1-200	1-100	1-100
TDCA	1	2	5-200	5-200	5-200
T-ω-MCA	50	100	100-2000	100-2000	100-2000
T-α-MCA	5	10	10-500	10-500	10-500
T-β-MCA	5	10	10-500	10-500	10-1000
GDHCA	0.5	1	/	/	/
TDHCA	0.5	1	/	/	/
CA-3S	0.5	0.5	1-100	1-100	1-100
CDCA-3S	0.5	0.5	1-500	1-100	1-100
UDCA-3S	1	1	1-500	1-100	1-100
LCA-3S	1	1	1-500	1-100	1-100
DCA-3S	0.5	0.5	1-500	1-100	1-200
GCA-3S	0.5	1	1-100	1-100	1-100
TCA-3S	1	1	1-100	1-100	1-200
GCDCA-3S	2	2	5-200	5-200	5-200
TCDCA-3S	1	1	1-100	1-100	1-100
GUDCA-3S	0.5	1	1-100	1-100	1-100
TUDCA-3S	1	2	5-200	5-200	5-200
GLCA-3S	2	2	5-200	5-200	5-200
TLCA-3S	2	5	5-200	5-200	5-200
GDCA-3S	1	1	1-100	1-100	1-100
TDCA-3S	1	1	1-100	1-100	1-100

Supplementary Table 12 Precision (%)

	serum			urine			feces		
	STD-L 10nmol/L	STD-M 100nmol/L	STD-H 1000nmol/L	STD-L 10nmol/L	STD-M 100nmol/L	STD-H 1000nmol/L	STD-L 10nmol/L	STD-M 100nmol/L	STD-H 1000nmol/L
CA	91.48	69.83	104.83	104.39	82.01	97.6	95.88	96.19	98.77
CDCA	99.72	112	96.5	121.23	118.55	101.74	115.53	114.62	101.23
α-MCA	115.69	84.27	97.27	111.92	111.53	92.7	105.79	101.89	97.91
β-MCA	134.08	109.44	98.65	91.44	113.79	102.8	94.7	103.35	99.07
HCA	51.33	46.8	91.17	145.53	104.58	102.02	95.02	108.57	103.78
HDCA	125.53	90.2	99.92	96.68	94.37	98	88.32	91.39	85.62
UDCA	153.12	90	105.05	111.76	76.95	92.7	84.28	106.36	101.85
LCA	103.01	103.83	97.65	111.25	107.46	99.44	114.32	107.41	99.34
7-ketoLCA	109.99	80.67	106.34	70.76	93.73	101.7	93.85	91.74	97.75
12-ketoLCA	117.3	102.37	116.75	90.01	107.68	101.27	148.13	100.03	97.89
DCA	84.3	117.32	106.9	138.56	119.59	98.27	121.11	110.1	102.31
23-norDCA	101.79	105.98	104.69	109.52	101.95	101.8	89.31	98.65	97.55
7-ketoDCA	112.57	114.18	98.27	109.26	104.77	99.86	97.15	96.85	92.66
3-ketoCA	111.01	115.27	91.65	85.12	92.14	90.81	86.57	109.82	99.95
dehydroLCA	108.02	104.5	103.21	106.79	95.95	96.8	101.9	101.03	97.91
allo-LCA	111.94	102.19	97.87	119.63	108.48	104.08	106.35	106.05	98.99
iso-LCA	109.81	100.28	96.28	112.47	106.96	101.5	108.33	102.13	100.2
6-ketoLCA	91.95	89.84	107.63	115.89	109.67	102.99	114.11	91.17	100.4
β-HDCA	78.36	103.2	106.71	99.64	117.34	103.2	78.53	114.25	104.06
β-UDCA	103.44	97.97	104.71	102.39	116.74	101.68	100.54	104.77	102.83
7,12-diketoLCA	113.66	101.65	94.76	82.98	107.52	98.54	91.53	106.94	96.09
6,7-diketoLCA	96.57	94.71	107.61	100.98	92.39	92.7	102.55	97.46	98.64
12-ketoCDCA	/	160.08	107.79	/	/	86.75	101.17	91.92	108.23
ω-MCA	129.86	97.42	108.4	116.08	97.21	108.67	96.56	112.14	99.67
β-CA	129.86	97.42	108.4	116.08	97.21	108.67	96.56	112.14	99.67
isoDCA	115.24	119.44	104.85	121.33	118.39	102.19	111.77	111.52	102.62
apoCA	91.77	91.87	108.3	112.9	116.67	101.46	107.71	110.12	103.81
muroCA	144.68	96.8	112.54	106.21	88.87	76.64	112.42	98.41	96.57
DHCA	115.37	117.69	111.81	114.77	114.81	101.95	117.33	113.35	99.56
UCA	120.94	119.32	103.95	95.97	81.61	101.63	108.49	106.86	108.58
ACA	/	95.32	92.07	88.86	98.31	99.56	121.95	95.61	93.97
GCA	113.93	108.52	103.51	107.98	104.59	100.74	106.47	102.43	97.55
TCA	104.98	99.18	95.07	123.44	99.02	93.86	112.5	103.74	96.69
GCDCA	122.59	102.59	104	111.97	102.07	105.31	115.91	105.89	94.55
TCDCA	82.91	105.81	90.82	95.83	110.7	89.04	87.11	113.5	99.37
GHCA	111.34	107.04	106.3	116.73	97.64	98.41	95.52	96.35	100.23
THCA	118.05	115.63	95.7	103.29	111.06	106.64	89.8	99.45	100.95
GHDCA	120.8	89.25	105.68	113.94	81.81	98.25	97.6	96.76	96.4
THDCA	99.52	119.11	100.88	91.93	108.09	100.98	97.12	98.21	98.43
GUDCA	116.68	83.44	92.57	115.02	69.11	97.46	105.9	84.3	85.29
TUDCA	111.81	108.34	100.27	85.32	93.51	85.39	114.26	103.09	101.09
GLCA	108.5	64.54	113.02	87.43	85.1	108.93	99.58	83.82	92.7
TLCA	78.09	104.48	94.63	111.6	111.22	98.74	68.73	94.89	97.28
GDCA	122.28	112.24	109.13	112.1	100.03	100.58	111.14	96.35	98.19
TDCA	104	113.02	94.37	96.85	117.43	107.37	105.94	103.58	97.4
T-ω-MCA	121.22	107.95	100.72	105.76	103.73	101.7	108.94	98.58	100.1
T-α-MCA	104.2	100.6	96.94	97.44	94.53	89.7	101.17	102.28	97.92
T-β-MCA	99.11	102.74	94.14	98.63	92.29	87.5	108.79	104.55	97.62
GDHCA	129.2	100.09	95.45	109.2	101.22	87.09	111.25	109.66	98.22
TDHCA	113.67	115.4	100.19	110.68	102.85	98.19	87.86	103.6	98.19
CA-3S	129.14	103.79	100.01	113.64	97.52	103.27	111.49	106.29	99.9
CDCA-3S	115.54	83.22	102.01	94.91	83.67	109.81	87.86	103.64	101.5
UDCA-3S	128.66	92.8	94.54	109.15	83.61	98.38	105.46	100.46	96.38
LCA-3S	124.38	80.16	102.51	97.28	65.88	98.29	123.01	101.37	100.38
DCA-3S	84.92	80.23	74.33	77.14	70.24	67.39	80.59	64.27	59.74
GCA-3S	117.03	107	100.3	105.67	98.49	97.83	90.33	101.06	99.65
TCA-3S	99.49	106.83	102.48	95.94	100.95	100.82	85.32	93.96	94.43
GCDCA-3S	98.27	100.16	100.72	93.47	103.64	96.6	/	98.04	97
TCDCA-3S	110.65	103.87	98.31	96.02	98.18	96.17	209.48	76.6	95.36
GUDCA-3S	114.45	107.33	99.58	116.98	107.93	98	111.66	91.88	99.61
TUDCA-3S	107.31	102.27	100.67	113.46	97.87	97.71	98.13	105.08	100.93
GLCA-3S	95.97	116.6	106.8	107.13	112.23	100.82	128.59	98.96	97.02
TLCA-3S	91.09	113.4	101.91	103.39	110.51	101.45	/	102.29	102.9
GDCA-3S	113.57	111.4	100.28	115.35	115.14	99.23	155.48	104.78	109.7
TDCA-3S	104.09	109.38	99.72	107.29	103.55	98.16	90.64	91.83	98.36

Supplementary Table 13 Intra-day accuracy (in RSD%)

	serum			urine			feces		
	STD-L 10nmol/L	STD-M 100nmol/L	STD-H 1000nmol/L	STD-L 10nmol/L	STD-M 100nmol/L	STD-H 1000nmol/L	STD-L 10nmol/L	STD-M 100nmol/L	STD-H 1000nmol/L
CA	12.65	4.21	1.19	8.45	2.97	4.23	7.88	3.03	4.34
CDCA	1.52	1.19	0.58	3	0.18	0.81	1.7	0.95	1.29
α-MCA	1.85	7.5	1.78	2.77	4.32	3.07	9.63	2.33	2.88
β-MCA	6.48	6.73	1.23	2.01	3.59	3.85	7.52	2.71	0.86
HCA	3.54	3.38	1.38	5.68	3.78	0.97	4.58	3.57	3.96
HDCA	8.7	7.02	1.05	13.4	4.38	2.13	5.54	7.86	0.82
UDCA	5.54	4.78	2.8	8.71	4.82	1.25	9.42	7.8	0.78
LCA	1.54	1.99	0.98	2.84	1.58	0.64	1.97	1.77	1.42
7-ketoLCA	15.41	9.82	1.95	6.01	14.3	1.76	13.95	8.01	0.99
12-ketoLCA	9.48	3.69	3.02	9.75	5.56	1.05	12.31	4.31	3.06
DCA	0.65	0.73	0.58	2.32	0.59	0.35	1.01	1.1	1.2
23-norDCA	12.7	10.79	2.52	4.88	4.73	3.35	17.95	6.45	3.18
7-ketoDCA	2.6	5.72	0.9	4.67	3.27	3.84	6.54	0.96	0.77
3-ketoCA	1.98	2.49	2.64	9.86	2.25	2.64	7.72	4.7	3.03
dehydroLCA	6.32	1.89	1.98	3.28	1.29	0.71	3.48	0.82	1.57
allo-LCA	2.17	1.15	2.05	2.88	1.4	0.54	1.66	0.97	0.98
iso-LCA	4.31	0.83	1.64	3.31	0.36	0.87	1.88	3.42	5.23
6-ketoLCA	4.88	6.14	1.78	7.54	11.35	6.29	35.3	9.06	2.7
β-HDCA	17.39	16.5	1.51	19.7	9.4	0.81	4.64	6.98	0.3
β-UDCA	7.32	7.29	5.49	10.42	5.1	2.94	6.44	6.31	1.03
7,12-diketoLCA	9.07	3.54	2.74	2.86	6.76	3.55	7.94	6.48	1.36
6,7-diketoLCA	12.79	3.14	0.95	8.16	5.22	3.96	6.62	10.68	2.75
12-ketoCDCA	16.48	10.18	3.73	10.36	6.57	6.29	/	24.52	4.3
ω-MCA	2.01	1.25	0.56	3.44	2.39	1.61	5.02	2.52	2.73
β-CA	2.01	1.25	0.56	3.44	2.39	1.61	5.02	2.52	2.73
isoDCA	2.25	0.42	0.48	2.92	1.17	0.67	1.39	1.11	1.15
apoCA	13.46	5.96	3.84	34.58	6.08	3.16	6.03	9.52	1.46
muroCA	9.84	13.17	4.68	26.14	5.84	1.93	7.64	6.32	3.67
DHCA	5.57	8.98	2	2.03	3.09	2.88	15.16	5.9	3.33
UCA	8.4	2.93	1.37	4.35	5.07	1.72	7.3	3.65	2.71
ACA	8.47	3.18	1.62	7.72	7.56	3.83	4.54	9.77	3.92
GCA	5.91	1.39	0.88	8.3	6.36	1.83	17.39	6.38	1.53
TCA	3.4	2.97	3.6	4.63	4.38	1.68	7.7	6.47	2.35
GCDCA	8.03	3.73	2.06	12.55	3.27	1.13	4.74	4.26	0.91
TCDCA	8.76	0.84	1.12	2.43	2.64	2.01	6.06	7.22	4.37
GHCA	4.13	4	1.6	12.79	4.23	1.65	16.67	6.34	2.78
THCA	2.7	2.59	0.94	5.36	1.96	1.12	5.99	5.92	4.16
GHDCA	2.21	4.42	2.19	7.51	6.67	1.92	16.93	10.28	3.32
THDCA	4.04	1.46	2.24	3.71	3.93	1.28	7.34	4.63	0.75
GUDCA	4.9	6.01	3.01	6.78	4.2	1.4	6.47	2.81	2.5
TUDCA	6.8	7.42	1.91	9	1.6	3.16	6.7	3.81	3.07
GLCA	11.29	4.3	2.44	8.45	5.25	0.99	4.96	2.9	1.62
TLCA	44.17	2.62	1.1	13.71	7.14	3.38	9.04	6.22	1.76
GDCA	4.74	0.7	1.11	4.76	1.77	1.54	5.4	2.14	3.73
TDCA	4.91	1.3	1.36	5	0.63	1.84	4.2	1.76	3.03
T-ω-MCA	5.01	8.6	3.72	6.34	3.63	2.67	9.59	2.78	3.66
T-α-MCA	4.49	1.55	1.26	8.66	2.73	1.48	11.24	7.69	2.93
T-β-MCA	4.96	3.44	3.86	6.7	4.27	2.66	8.92	7.6	1.44
GDHCA	13.51	1.88	1.22	12.19	3.25	0.78	11.54	6.27	2.97
TDHCA	9.74	1.84	1.15	7.64	5.1	0.71	9.61	4.99	0.85
CA-3S	9.29	1.79	0.78	4.93	1.07	0.71	9.7	3.26	1.08
CDCA-3S	5.84	2.22	2.08	7.01	3.41	2.68	5.32	6.42	0.8
UDCA-3S	5.02	2.09	0.73	8.77	2.65	1.45	5.83	1.23	1.24
LCA-3S	9.79	2.37	2	7.25	7.19	2.8	8.3	2.83	2.56
DCA-3S	5.9	3.38	4.06	2.91	1.58	0.68	4.07	2.56	3.06
GCA-3S	6.11	2.01	1.32	5.6	4.41	0.86	11.55	4.07	2.14
TCA-3S	14.32	1.14	1.64	4.71	3.97	1.71	17.72	12.53	5.89
GCDCA-3S	17.5	4.14	1.69	14.95	5.75	0.65	5.86	6.05	1.46
TCDCA-3S	23.87	3.56	3.55	9.18	1	2.34	/	2.86	3.15
GUDCA-3S	7.56	5.28	1.23	7.59	4.55	1.08	7.16	2.05	3.82
TUDCA-3S	4.53	3.61	1.21	8.27	3.65	2.81	14.9	1.69	4.39
GLCA-3S	43.99	7.45	1.48	28.87	5.28	1.29	13.71	1.22	2.56
TLCA-3S	27.3	2.72	3.54	9.02	7.52	3.29	/	8.31	6.69
GDCA-3S	6.46	7.12	2.33	9.42	4.33	1.91	23.67	9.41	4.59
TDCA-3S	11.82	1.36	1.63	6.17	3.06	0.43	13.13	6.8	3.14

Supplementary Table 14 Inter-day accuracy (in RSD%)

	serum			urine			feces		
	STD-L 10nmol/L	STD-M 100nmol/L	STD-H 1000nmol/L	STD-L 10nmol/L	STD-M 100nmol/L	STD-H 1000nmol/L	STD-L 10nmol/L	STD-M 100nmol/L	STD-H 1000nmol/L
CA	9.61	5.01	3.74	8.69	4.93	6.48	8.89	4.24	4.13
CDCA	2.16	3.45	2.12	3.44	3.21	0.72	1.54	1.71	2.27
α-MCA	4.58	5.28	2.24	8.91	5.57	2.63	7.75	5.64	4.48
β-MCA	7.84	4.48	1.44	3.27	3.17	2.95	5.17	2.34	4.05
HCA	4.17	4.53	2.8	4.45	2.51	2.08	4.18	3.36	6.66
HDCA	8.06	6.85	2.71	9.35	5.87	2.41	5.6	5.59	1.45
UDCA	9.00	6.37	2.69	8.88	8.32	1.86	12.29	7.87	2.28
LCA	4.44	4.96	1.19	3.3	3.3	0.89	2.84	2.02	2.28
7-ketoLCA	19.11	10.27	2.09	12.92	7.42	4.78	11.04	11.72	1.26
12-ketoLCA	15.49	8.82	3.26	13.08	6.79	1.76	13.25	8.24	3.82
DCA	4.08	3.18	1.59	1.89	2.64	1.05	3.54	2.38	2.07
23-norDCA	20.35	11.95	3.24	12.96	9.69	5.92	18.07	9.04	3.15
7-ketoDCA	4.59	4.73	1	7.83	4.22	3.98	7.65	1.98	1.17
3-ketoCA	14.11	3.66	2.09	5.91	4.54	2.61	10.48	4.25	4.99
dehydroLCA	7.03	3.85	1.93	2.92	3.15	0.86	6.99	4.26	5.95
allo-LCA	3.74	4.25	1.7	3.2	2.46	0.77	2.86	2.14	2.96
iso-LCA	4.26	4.06	1.39	3.52	3.06	0.81	2.86	3.87	3.74
6-ketoLCA	16.28	5.93	4.58	13.02	8.73	4.78	28.15	8.69	3.32
β-HDCA	15.67	12.49	1.58	14.31	7.47	2.17	13.76	5.68	3.14
β-UDCA	7.56	5.2	3.94	13.27	7.72	2.63	11.98	9.27	1.5
7,12-diketoLCA	9.44	4.76	2.91	10.67	5.81	4.7	13.96	4.07	1.61
6,7-diketoLCA	11.63	8.42	0.93	8.35	9.17	4.04	6.55	6.81	2.98
12-ketoCDCA	11.42	7.97	6.01	16.74	10.1	6.12	23.49	19.97	3.9
ω-MCA	6.96	1.41	1.18	9.68	3.07	1.53	4.83	3.87	3.67
β-CA	6.96	1.41	1.18	9.68	3.07	1.53	4.83	3.87	3.67
isoDCA	3.8	1.25	1.37	2.55	1.96	0.73	2.24	0.99	2.8
apoCA	17.58	11.27	3.75	12.12	8.36	3.33	16.24	6.65	1.81
muroCA	19.34	8.49	4.52	8.39	4.59	2.33	9.76	10.48	5.89
DHCA	12.42	9.46	1.81	9.44	6.85	2.54	14.12	6.04	3.83
UCA	7.98	5.74	1.56	6.17	7.09	3.16	6.12	3.97	5.46
ACA	9.88	3.63	3.43	11.39	8.87	4.2	9.6	11.69	6.08
GCA	14.68	2.48	1.95	9.99	5.52	1.47	13.11	5.18	1.6
TCA	5.53	3.3	3.96	5.03	4.26	3.66	6.53	8.24	3.2
GCDCA	7.64	3.92	2.03	11.67	2.92	1.44	5.63	4.09	3.4
TCDCA	10.94	5.93	1.38	10.48	3.02	2.22	9.93	5.68	3.63
GHCA	9.8	6.86	2.42	11.83	6.73	1.79	14.72	6.59	3.05
THCA	11.12	2.74	1.89	4.8	2.05	2.33	6.73	5.53	3.45
GHDCA	7.85	5.22	3.55	7.55	6.58	2.56	12.03	10.28	4.73
THDCA	7.8	4.32	2.48	3.9	5.34	1.26	7.33	8.51	2.03
GUDCA	6.32	5.02	2.71	4.75	6.21	2.37	4.95	3.06	3.59
TUDCA	10.69	6.99	3.31	7.34	4.07	2.89	6.52	3.63	2.99
GLCA	12.46	3.66	1.99	12.37	5.66	2.71	4.51	4.95	1.69
TLCA	14.9	5.75	1.16	9.86	5.39	4.6	12.58	4.84	2.97
GDCA	8.67	3.36	1.85	3.78	2.68	1.07	4.91	3.61	2.8
TDCA	4.45	1.12	1.75	4.86	3.21	1.93	10.43	3.07	2.79
T-ω-MCA	4.58	5.93	3.28	7.45	4.52	2.5	8.08	7.67	2.9
T-α-MCA	5.85	1.58	1.24	9.41	5.33	2.23	12.51	6.2	2.4
T-β-MCA	4.59	4.07	2.97	5.84	4.66	2.25	7.25	7.18	2.34
GDHCA	18.72	4.47	1.06	12.95	2.55	1.09	17.85	8.63	3.12
TDHCA	7.59	3.78	1.47	13.17	5.04	1.35	10.1	4.41	1.58
CA-3S	8.93	3.64	0.96	6.24	1.59	0.49	7.51	2.81	2.69
CDCA-3S	5.5	2.06	2	5.12	2.36	2.37	6.3	6.64	3.03
UDCA-3S	4.8	1.31	0.82	11.67	2.52	1.32	8.35	1.49	1.5
LCA-3S	9.04	4.45	2.54	13.8	8	3.38	9.25	5.5	2.78
DCA-3S	4.89	3.64	4.97	3.33	4.07	2.09	5.66	2.84	2.83
GCA-3S	14.72	4.21	1.72	7.19	3.61	1.42	12.87	5.06	2.14
TCA-3S	10.06	4.73	2.59	5.77	4.42	3.07	11.82	9.66	4.87
GCDCA-3S	10.14	5.65	1.36	12.84	7.49	1.69	17.17	5.19	1.98
TCDCA-3S	15.55	3.86	3.82	13.9	3.6	2.69	45.02	6.02	4.09
GUDCA-3S	10.27	6.16	1.6	12.58	4.3	1.07	13.47	8.75	3.08
TUDCA-3S	9.09	3.02	1.34	7.67	4.16	3.6	14.2	4.95	3.59
GLCA-3S	7.48	6.36	1.72	13.53	5.86	1.84	8.62	3.23	2.21
TLCA-3S	17.84	2.92	3.53	12.29	9.93	4.82	28.34	13.66	5.42
GDCA-3S	7.09	5.52	3.59	12.16	4.03	2.38	17.61	8.24	3.19
TDCA-3S	11.54	1.14	1.7	9.55	2.74	1.07	19.61	10.51	2.42

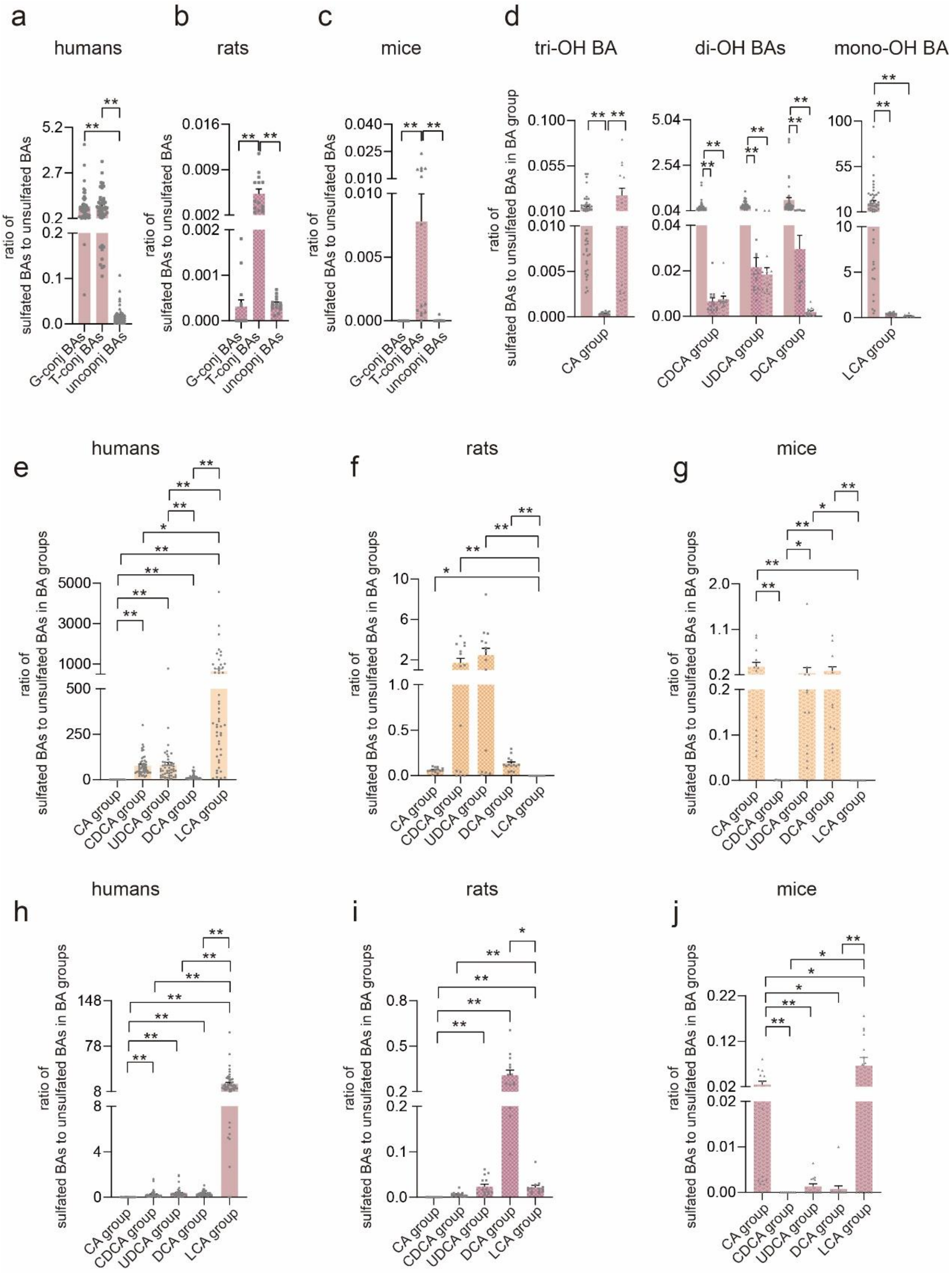

Supplementary Figure 1. **The ratio of sulfated BAs to BAs**. **a-c** The ratio of sulfated BAs to unsulfated BAs in humans, rats and mice urine. **d** The ratio of sulfated BA to unsulfated BA in BA groups among humans, rats, and mice urine. **e-g** The ratio of sulfated BA to unsulfated BA in humans, rats and mice urine. **h-j** The ratio of serum sulfated BA to unsulfated BA in humans, rats and mice serum. G-conj-BAs: glycine-conjugated BAs, T-conj-BAs: taurine-conjugated BAs, unconj-BAs: unconjugated BAs, tri-OH BAs:

trihydroxy BA, di-OH BA: dihydroxy BA, mono-OH BAs: monohydroxy BAs. CA group: the sum of CA, TCA and GCA. DCA group: the sum of DCA, TDCA and GDCA. CDCA group: the sum of CDCA, TCDCA and GCDCA. LCA group: LCA, TLCA and GLCA. * indicates $p < 0.05$, ** indicates $p < 0.01$. Error bars depict the standard error of the mean (SEM).

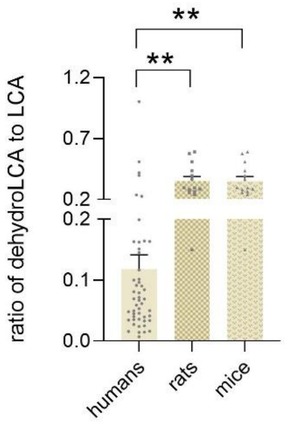

Supplementary Figure 2 The ratio of dehydroLCA to LCA in feces. Data are presented as the mean ± SEM. * indicates $p < 0.05$, ** indicates $p < 0.01$. Error bars depict the standard error of the mean (SEM).
